# Host-to-Pathogen Gene Transfer Facilitated Infection of Insects by a Pathogenic Fungus

**DOI:** 10.1371/journal.ppat.1004009

**Published:** 2014-04-10

**Authors:** Hong Zhao, Chuan Xu, Hsiao-Ling Lu, Xiaoxuan Chen, Raymond J. St. Leger, Weiguo Fang

**Affiliations:** 1 Institute of Microbiology, College of Life Science, Zhejiang University, Hangzhou, China; 2 Department of Entomology, University of Maryland, College Park, Maryland, United States of America; Geisel School of Medicine at Dartmouth, United States of America

## Abstract

*Metarhizium robertsii* is a plant root colonizing fungus that is also an insect pathogen. Its entomopathogenicity is a characteristic that was acquired during evolution from a plant endophyte ancestor. This transition provides a novel perspective on how new functional mechanisms important for host switching and virulence have evolved. From a random T-DNA insertion library, we obtained a pathogenicity defective mutant that resulted from the disruption of a sterol carrier gene (*Mr-npc2a*). Phylogenetic analysis revealed that *Metarhizium* acquired *Mr-npc2a* from an insect by horizontal gene transfer (HGT). Mr-NPC2a binds to cholesterol, an animal sterol, rather than the fungal sterol ergosterol, indicating it retains the specificity of insect NPC2 proteins. Mr-NPC2a is an intracellular protein and is exclusively expressed in the hemolymph of living insects. The disruption of *Mr-npc2a* reduced the amount of sterol in cell membranes of the yeast-like hyphal bodies that facilitate dispersal in the host body. These were consequently more susceptible to insect immune responses than the wild type. Transgenic expression of Mr-NPC2a increased the virulence of *Beauveria bassiana*, an endophytic insect-pathogenic fungus that lacks a Mr-NPC2a homolog.

## Author Summary

The ability of infectious agents to evolve different host ranges contributes to the emergence of new diseases, and this host switching could also account for the wide variety of fungal associations with animals, plants and other fungi. There must be mechanisms for such host shifts, but these remain largely unknown. In this study, we phylogenetically predict that the endophytic fungus *Metarhizium robertsii* acquired a sterol carrier gene from insects through horizontal gene transfer (HGT). This sterol carrier is involved in maintaining cell membrane sterols, and thus membrane integrity, when *M. robertsii* proliferates in the haemocoel of living insects. Therefore, the acquisition of genetic material from a host has contributed to the development of fungal entomopathogenicity. In order to simulate this evolutionary event, the sterol carrier gene was transformed into an endophytic insect-pathogenic fungus (*Beauveria bassiana*) that lacks an endogenous Mr-NPC2a homolog. The virulence of *B. bassiana* was increased by expression of Mr-NPC2a.

## Introduction

New infectious diseases are constantly appearing, and their origins are diverse. Human behavior and practices are important factors, for example, catastrophic declines in amphibian and bat populations have been attributed to pathogenic fungi spread by humans [1], [2]. Another important origin is the ability of infectious agents themselves to evolve different host ranges, which would certainly contribute to the impact of invasive species. Such host switching probably accounts for the wide variety of fungal associations with animals, plants and other fungi [3]. There must be mechanisms for such host shifts, although these remain largely unknown [4], [5], [6].

Horizontal gene transfer (HGT) between distantly related bacteria contributes significantly to the emergence of new pathogens but HGT is usually thought to play a minor role in eukaryotes. However, data from multiple genomic sequences suggests that HGT has also occurred between eukaryotes [7], and may bestow a clear selective advantage to fungi [8]. A gene encoding a critical virulence factor was transferred from one species of fungal pathogen to another, leading to the emergence of a new damaging disease of wheat [9]. HGT from bacteria to fungi is relatively common [10], and a few examples are known where HGT has occurred between plants and either pathogenic fungi or parasitic plants [11], [12], [13], [14]. Microsporidian intracellular parasites *Enephalitozoon* may have acquired a purine nucleotide phosphorylase from an arthropod that is not a host [15]. There is also a longstanding controversy as to whether the malaria causing pathogen *Plasmodium vivax* has horizontally acquired human genetic material that might facilitate its long stay in the body [16]. Otherwise, HGT between eukaryotic pathogens and animal hosts has been neither predicted nor characterized.

Fungi are the commonest pathogens of insects and crucial regulators of insect populations [17], [18]. *Metarhizium robertsii* (formerly known as *Metarhizium anisopliae*) is the best studied entomopathogenic fungus [19] as well as being an important plant-growth-promoting endophyte [20], [21]. Phylogenomic analysis suggests that the ancestor of *Metarhizium* was an endophyte, with entomopathogenicity being an acquired characteristic [22]. The evolutionary transition of a fungus from an endophyte to an insect pathogen provides a novel perspective on how new functional mechanisms important for host switching and virulence are acquired. In this study, we identified a new virulence factor (Mr-NPC2a) of *M. robertsii* by screening an *Agrobacterium tumefaciens*-mediated T-DNA insertion library. We provide evidence that *Mr-npc2a* was horizontally acquired from an insect and allowed *Metarhizium* to compete with insect hosts for the sterols necessary to maintain cell membrane integrity.

## Results

### Characterization of the sterol carrier gene *Mr-npc2a* as a pathogenicity mutant

**Figure 1:**
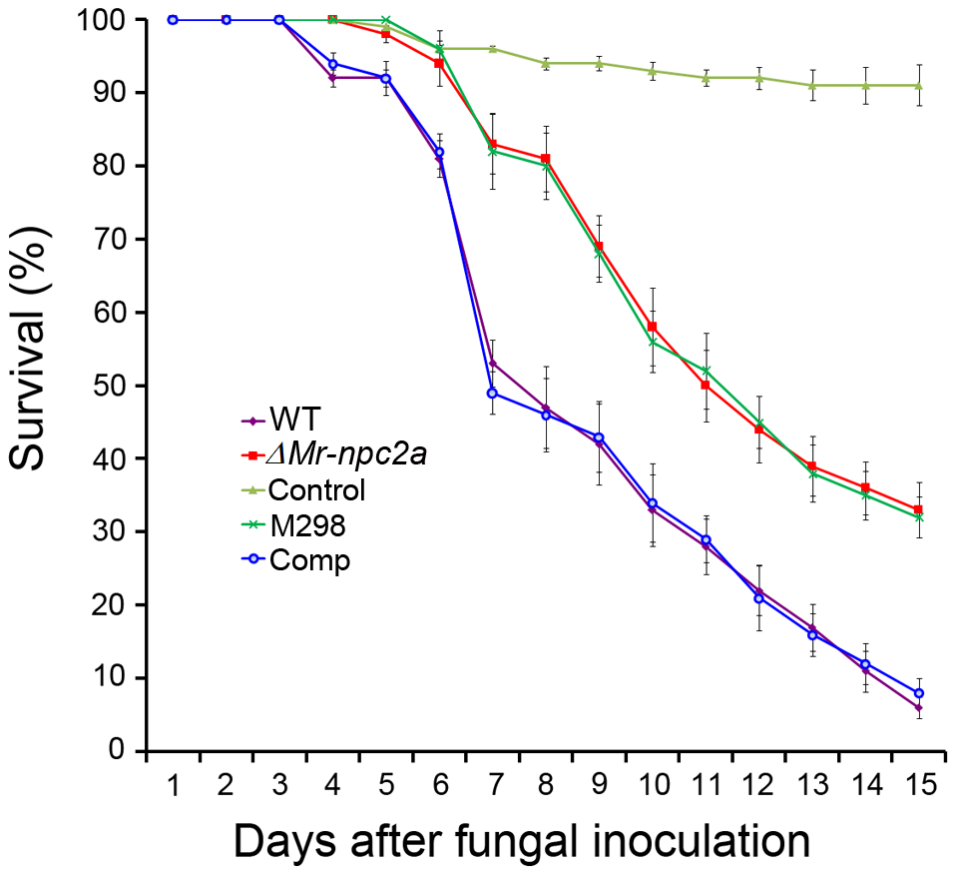
Kinetics of insect survivorship in bioassays. Wax worm larvae (*G. mellonella*) were treated with *M. robertsii* conidial suspensions (1×10^7^ spores/mL). WT: the wild type strain; *ΔMr-npc2a*: the mutant with the ORF of *Mr-npc2a* deleted; M298: the mutant with *Mr-npc2a* disrupted by T-DNA insertion; Comp: the complemented *ΔMr-npc2a* with a genomic clone of *Mr-npc2a*; control: insects treated with 0.05% Tween-80.

Using *Agrobacterium tumefaciens*-mediated fungal transformation, we generated a set of 20, 328 T-DNA-tagged *M. robertsii* strain ARSEF2575 mutants [23] which we screened for virulence mutants against wax worm larvae (*Galleria mellonella*) (Pet Solutions, OH USA). A transformant (M298) that showed a significantly slower killing speed than the wild type strain was selected for further study (Fig. 1). The open reading frame (ORF) of the gene MAA_03817 (Genbank accession no.: EFZ01221) was found to be disrupted in this mutant. MAA_03817 showed significant similarity to insect Niemann-Pick type C2 (NPC2) proteins, and thus was designated as Mr-NPC2a. We deleted the ORF of *Mr-npc2a* in wild type *M. robertsii*, and the resulting disruptant (*ΔMr-npc2a*) showed the same loss of virulence as M298 (Fig. 1). The details of the T-DNA insertion and gene deletion alleles are shown in Fig. S1. *ΔMr-npc2a* was complemented by a genomic clone of *Mr-npc2a*. The details of deletion of *Mr-npc2a* and the complementation of *ΔMr-npc2a* are described in Fig. S2.

The phenotype of *ΔMr-npc2a* on PDA (potato dextrose agar) plates was indistinguishable from the wild type strain, M298 and the complemented *ΔMr-npc2a*. Formation of infection structures (appressoria) by *ΔMr-npc2a* on the hindwings of *Locusta migratoria manilensis* (kindly provided by Dr. Wangpeng Shi at China Agricultural University) was not significantly different from the wild type strain, M298 and the complemented *ΔMr-npc2a*. The hyphal bodies [unicellular hyphal bodies (blastospores) and multicellular hyphal bodies] of *ΔMr-npc2a*, M298, the wild type strain and the complemented *ΔMr-npc2a* all began to appear in the hemolymph 36-hours after topical infection of *G. mellonella*, but the number of *ΔMr-npc2a* hyphal bodies (20±6.8 hyphal bodies/mL) and M298 (22±3.2 hyphal bodies/mL) was significantly less than the wild type (36 hyphal bodies ±9.3/mL) and the complemented *ΔMr-npc2a* (38 hyphal bodies ±9.5/mL). From 36 to 60 h post-inoculation, wild type hyphal bodies and the complemented *ΔMr-npc2a* hyphal bodies significantly outnumbered *ΔMr-npc2a* and M298 in the hemolymph. The number of the complemented *ΔMr-npc2a* hyphal bodies was not significantly different from the wild type strain at all time points (*P*>0.05). Likewise, the number of M298 hyphal bodies was not significantly different from *ΔMr-npc2a* at all time points (*P*>0.05) (Table S1). This set of data suggests that disruption of *Mr-npc2a* only impairs pathogenesis after *M. robertsii* enters the haemocoel.

All *Galleria* infected with the wild type strain or the complemented *ΔMr-npc2a* died as larvae, whereas nearly 40% of insects infected by *ΔMr-npc2a* or M298 survived. Similar numbers of conidia were produced on the cadavers of insects killed by the wild type strain, by the complemented *ΔMr-npc2a*, by *ΔMr-npc2a* and by M298 (*P*>0.05), so in terms of fecundity Mr-NPC2a enhanced fitness by 40%. Based on above data, M298 and *ΔMr-npc2a* are functionally indistinguishable, so only *ΔMr-npc2a* was used for following studies.

### *Metarhizium* acquired the sterol carrier Mr-NPC2a from insect hosts by horizontal gene transfer

Genome analysis showed that there are 3 NPC2-like proteins in *M. robertsii*: MAA_03817 (Mr-NPC2a), MAA_10401 (Mr-NPC2b, Genbank accession no.: EFY94139) and MAA_03340 (Mr-NPC2c, Genbank accession no.: EFZ00744). All three *Metarhizium* NPC2-like proteins have conserved putative cholesterol/lipid binding sites, and six cysteine residues that form a hydrophobic core for sterol binding typical of Niemann-Pick type C2 (NPC2) sterol carriers. Mr-NPC2b shows 70% similarity to Mr-NPC2c (6e^−53^), but except for the aforementioned cholesterol/lipid binding sites and hydrophobic core, they show no significant similarity to Mr-NPC2a. We screened for homologs of Mr-NPC2a, Mr-NPC2b and Mr-NPC2c proteins using a protein based local alignment search tool (BLASTP, e-value cutoff 1e^−05^) against databases provided by NCBI (Genbank), JGI (Joint Genome Institute) and Broad Institute. No Mr-NPC2a homologs were identified when Mr-NPC2b or Mr-NPC2c were used as the query sequences; likewise no Mr-NPC2b and Mr-NPC2c homologs were identified when Mr-NPC2a was used as the query. Thus, BLASTP clearly differentiates between the evolutionary relationships of Mr-NPC2a and the related Mr-NPC2b and Mr-NPC2c. Homologs of Mr-NPC2b and Mr-NPC2c are widespread in the fungal kingdom being found in Ascomycota, Basidomycota, Zygomycota and Chytridiomycota. A phylogenetic analysis conducted with Mr-NPC2b, Mr-NPC2c and 30 other NPC2s showed that their phylogenetic relationship is consistent with previously established species phylogenies, demonstrating vertical inheritance (Fig. S3).

**Figure 2:**
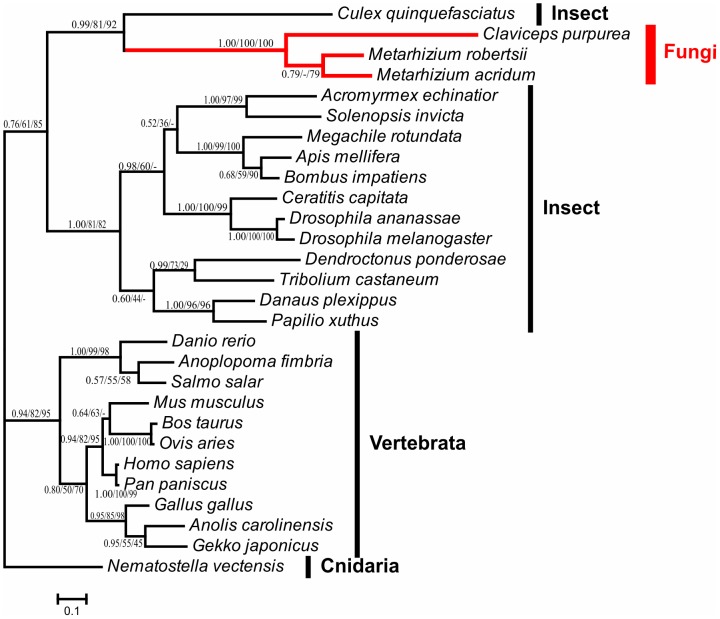
Phylogenetic analysis of fungal NPC2a proteins and their homologs. The Bayesian inference tree is shown unrooted. Numbers at nodes represent bayesian posterior probabilities (left) and bootstrap values of maximum likelihood (middle) and neighbor-joining (right) respectively. Hyphen (-) indicates no support value in corresponding method. The scale bar corresponds to the estimated number of amino acid substitutions per site. Branches of *Clavicipitaceae* (fungi) are indicated in red. The information about the sequences used in this analysis is presented in Table S2.

Mr-NPC2a homologs were identified in only two fungi (BLASTP, e-value cutoff 1e^−05^)–the ergot fungus *Claviceps purpurea* and *Metarhizium acridum*. All other Mr-NPC2a homologs were from insects and other animals including vertebrates. No homologs were found in plant, bacteria, archaea and viruses. A TBLASTn search against NCBI genomes (e-value cutoff 1e^−05^) only identified the same homologs as BLASTP. The origin of NPC2a was assessed using a variety of models and methods for phylogenetic reconstruction. The phylogenies consistently showed that *Metarhizium* and *C. purpurea* sequences exclusively clustered with insect sequences with high support (Fig. 2, Table S2). We then compared the topology of the obtained tree with those of alternative trees using eight tests including SH-test and AU-test provided by the program CONSEL [24]. The cluster of fungal NPC2as with vertebrate NPC2s was not statistically rejected, but these constrained trees were significantly less supported than the obtained tree. Furthermore, vertebrates lack close contact with *Metarhizium* and *C. purpurea*, whereas insects regularly encounter both fungi [17], [25]. Since sympatry is almost a prerequisite for HGT [26], the fungi are much more likely to have acquired NPC2a from insects than vertebrates. Details about the tree topology comparisons are presented in Table S3.

Although it is the most likely that *Metarhizium* and *C. purpurea* acquired NPC2a from insects, the fungal NPC2as show low overall similarity to insect sequences (the lowest E value was 6e^−15^). However, NPC2 paralogs are remarkably divergent even in the same insect species, consistent with rapid diversification. For example, the fruitfly *Drosophila melanogaster* has 8 NPC2 proteins designated as NPC2a-NPC2h (Genbank accession numbers are NP_608637, NP_650331, NP_649976, NP_649975, NP_731439, NP_651219, NP_651823 and NP_651824, respectively), and the similarity of *D. melanogaster* NPC2a to other NPC2 proteins ranges from 3e^−16^ (NPC2b, 50% similarity) to as low as 3e^−03^ (NPC2g, 37% similarity). It is likely therefore that the insect ancestor of Mr-NPC2a could also have had low similarity to currently known insect NPC2 proteins.

**Figure 3:**
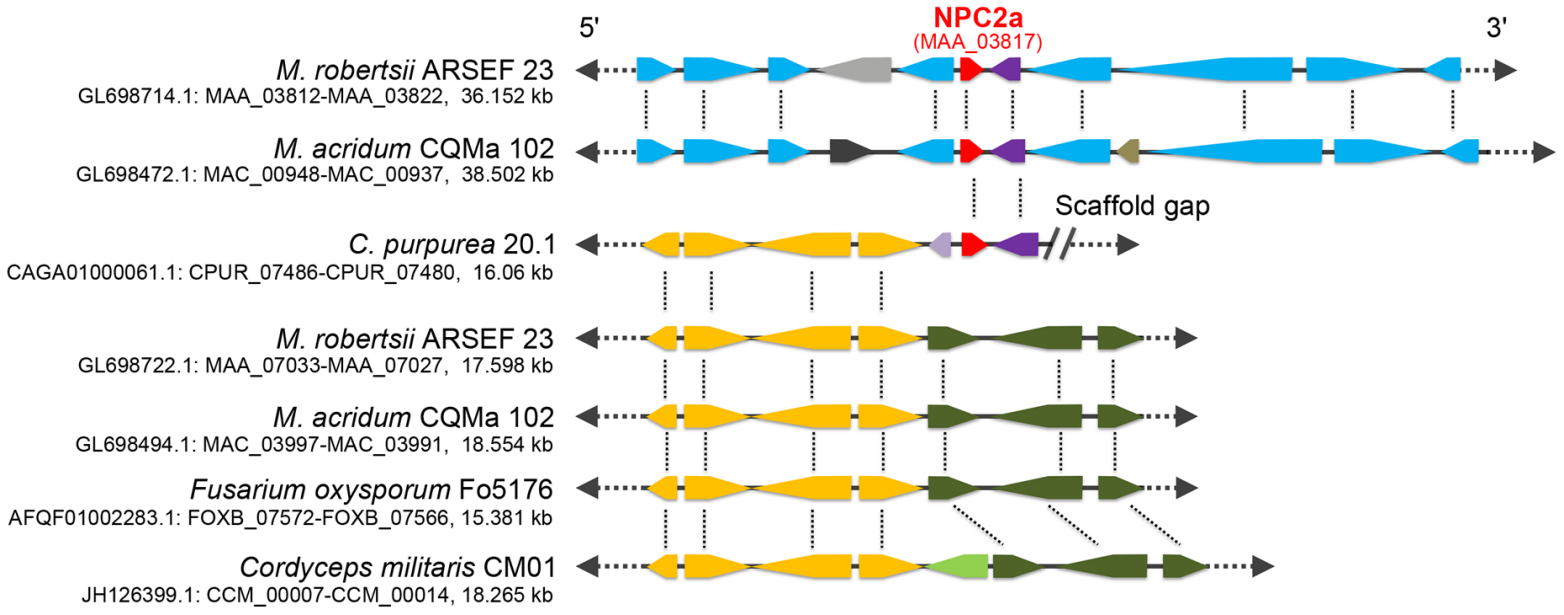
Genomic context of the gene *npc2a* in *M. robertsii*, *M. acridum*, *C. purpurea*, and 16 other fungi (represented by *Fusarium oxysporum* and *Cordyceps militaris*). Homologs are shown in the same color. Red: *npc2a* gene. Purple: a gene that is adjacent to *npc2a* and is conserved among in *M. robertsii*, *M. acridum* and *C. purpurea*. Blue: genes are homologous between *M. robertsii* and *M. acridum*; Yellow: Genes that are co-linear in *C. purpurea*, *M. robertsii*, *M. acridum*, *F. oxysporum* and *C. militaris*. Dark olive: genes that are co-linear in *M. robertsii*, *M. acridum*, *F. oxysporum* and *C. militaris*. Note: a gap follows *C. purpurea npc2a* gene and the gene CPUR_07480.

We then looked at the genomic context of the *npc2a* genes in *Metarhizium* and *C. purpurea* to investigate how these fungi may have acquired *npc2a* gene from insects. The microsyntenies are almost the same in ∼40 kb *M. robertsii* and *M. acridum* DNA fragments containing the *npc2a* genes, indicating that the *npc2a* gene was acquired before the diversification of *M. robertsii* and *M. acridum* ∼33–43 MYA [22], and no significant genomic rearrangements have happened since. However, little microsynteny is retained between *Metarhizium* and other fungi including *C. purpurea*; only one *M. robertsii* gene (MAA_03818, Genbank accession no.: EFZ01222) adjacent to *Mr-npc2a* (MAA_03817) has a homolog (CPUR_07480, Genbank accession no.: CCE3355) adjacent to the *C. purpurea npc2a* gene (CPUR_07479, Genbank accession no.: CCE33554). CPUR_07480 and MAA_03818 have homologs in other fungi but not insects, showing that this gene is vertically inherited. CPUR_07480 and MAA_03818 have LITAF-like zinc ribbon domain, but their functions have not been characterized. Conversely, the microsynteny of the sequence around the *C. purpurea npc2a* gene (CPUR_07479) and CPUR_07480 is conserved among many fungi including *Metarhizium* (Fig. 3).

### The lipid binding activity of Mr-NPC2a

**Figure 4:**
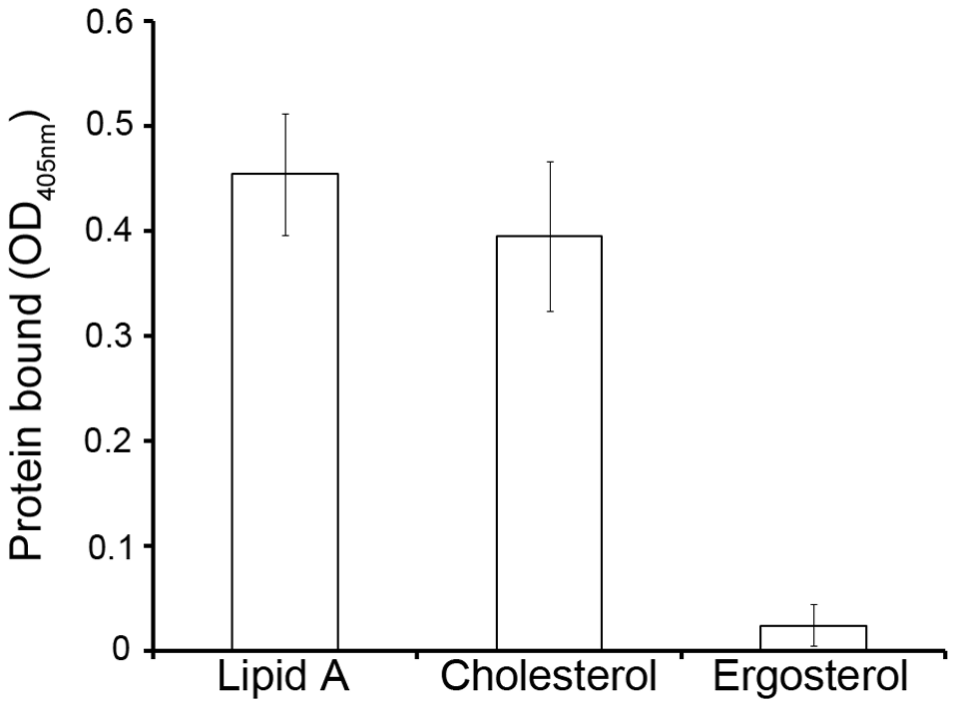
Binding of recombinant Mr-NPC2a to diphosphoryl lipid A from *E. coli*, cholesterol and ergosterol. Purified Mr-NPC2a fusion protein was diluted to 500 mM and added to the ligand-coated plates for ELISA assay. Each bar represents the mean of 3 individual measurements±SEM.

Mr-NPC2a was expressed in *Escherichia coli* using the pET-15 system. The recombinant protein was purified to homogeneity and subjected to a lipid binding assay. Mr-NPC2a binds to cholesterol and bacterial lipid A that are absent in fungi, but did not bind to fungal ergosterol (Fig. 4), indicating that its binding specificity was similar to *D. melanogaster* NPC2 proteins that binds to cholesterol and bacterial lipid A [27], and distinct from yeast yNPC2p that binds to ergosterol and is involved in ergosterol homeostasis [28].

### Expression pattern and cellular localization of Mr-NPC2a

**Figure 5:**
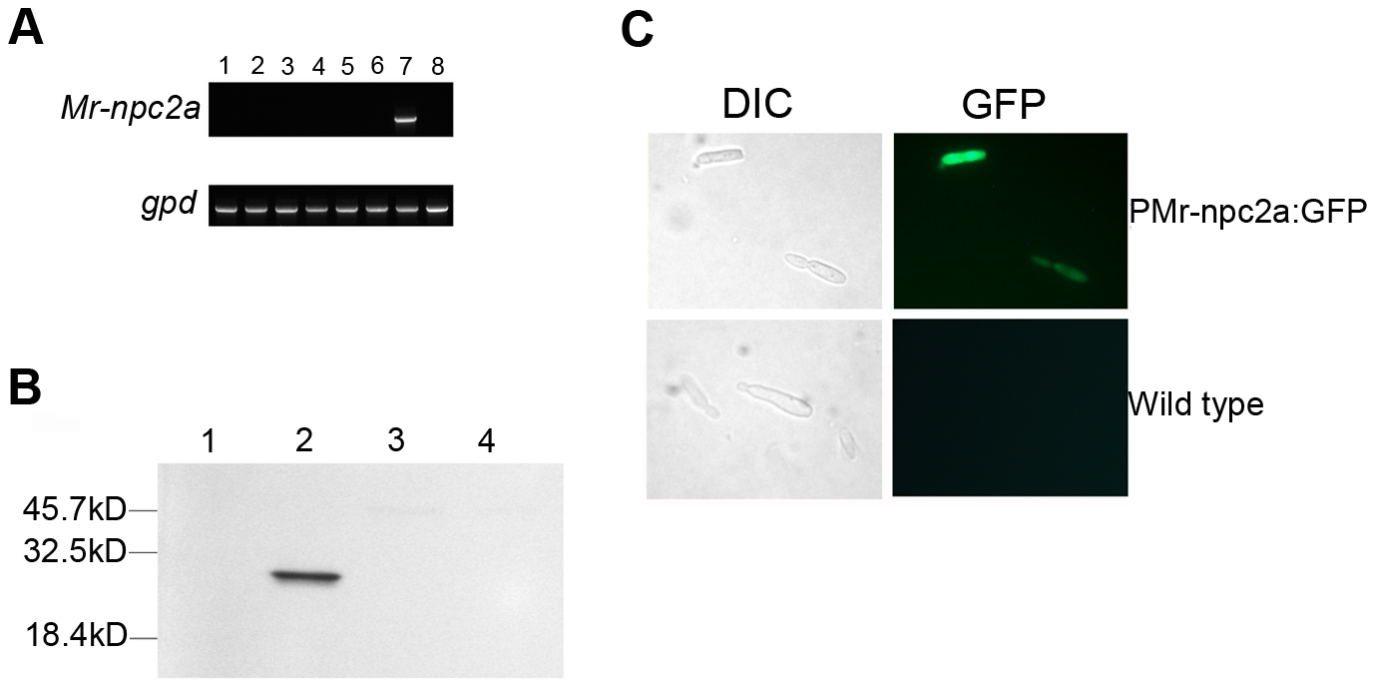
The expression pattern and cellular localization of Mr-NPC2a. (*A*) RT-PCR analysis of *Mr-npc2a* expression in the wild type strain. Top row: the expression of *Mr-npc2a* in 1: aerial hyphae collected from a PDA plate; 2: conidiating mycelium collected from a PDA plate which has hyphae, conidiphores and conidia; 3: conidia collected from a PDA plate; 4: mycelium collected from SDB broth which comprises of hyphae and blastospores; 5: appressoria formed on locust hindwings; 6: hyphae and blastospores grown in the *in vitro* prepared hemolymph; 7: Unicellular hyphal bodies (blastospores) and multicellular hyphal bodies collected from the hemolymph of living insects which also contains insect hemocytes; 8: insect hemocytes of healthy insects. Bottom row: 1 to 7 represents the expression of the gene *gpd* in the same samples described in the top row; 8 is the expression of ribosomal protein S3 gene (Genbank accession no. U12708) in the hemocytes of *M. sexta*. (*B*) Analysis of expression pattern of *Mr-npc2a* by following GFP signal in a transformant where GFP expression was driven by the promoter region of *Mr-npc2a*. GFP fluorescence only occurred in hyphal bodies in the hemolymph of living *M. sexta* larvae. PMr-npc2a:GFP: a transformant with *egfp* driven by the promoter region of *Mr-npc2a*; wild type: the wild type strain of *M. robertsii*; DIC: differential interference contrast; GFP: GFP fluorescence. (*C*) Localization of Mr-NPC2a. *M. robertsii* transformants expressing the fusion protein Mr-NPC2a: GFP driven by the constitutive *Pgpd* promoter were constructed to investigate the localization of Mr-NPC2a. A representative transformant was grown in SDB for 36 h, and total proteins in mycelium and supernatant were separately prepared for Western blot analysis using rabbit anti-GFP antibody. Total protein was prepared from mycelium of (1) wild type, (2) mycelium of a transformant expressing Mr-NPC2a:GFP, (3) supernatant of the wild type strain, and (4) supernatant of the transformant expressing Mr-NPC2a:GFP.

We then investigated the biological function of Mr-NPC2a in *M. robertsii*. RT-PCR (reverse transcription PCR) did not detect *Mr-npc2a* transcripts in aerial hyphae, conidiating mycelium and conidia collected from PDA plates, hyphae and blastospores collected from SDB (Sabouraud dextrose broth), hyphae and blastospores grown in the *in vitro* prepared insect hemolymph, and appressoria on locust hindwings. However, *Mr-npc2a* was transcribed in the hyphal bodies collected from the hemolymph of living insects (Fig. 5*A*). The expression pattern of Mr-NPC2a was also investigated by observing GFP signal in three randomly selected transformants containing PMr-NPC2a:GFP. No differences in GFP signal at different developmental stages were observed between these transformants, so only data about one of the transformants is shown. GFP fluorescence was only observed in both unicellular (blastospores) and multicellular hyphal bodies after the fungus had penetrated the insect host (*Manduca sexta* larva) (Carolina, Burlington, NC) cuticle (Fig. 5*B*). Therefore, both RT-PCR analysis and GFP observation showed that Mr-NPC2a expresses exclusively in the hemolymph of living insects irrespective of whether the hyphal bodies were unicellular (blastospores) or multicellular. This pattern differs from other hemolymph specific promoters such as Mcl1 [29], as these are also active in *in vitro* prepared hemolymph.

We tried to identify the hemolymph inducers of *Mr-npc2a* expression, but found sterols, an insect steroid hormone (20-Hydroxyecdysone), oxidative stress, hypoxia stress, osmotic stress did not trigger the expression of *Mr-npc2a* (Table S4).

In order to investigate the cellular distribution of Mr-NPC2a we placed the fusion protein Mr-NPC2a:GFP under control of the constitutive glyceraldehyde-3-phosphate dehydrogenase (*gpd*) promoter from *Aspergillus nidulans* [30]. No GFP was observed in the hyphae of the transformants, but Western blotting analysis detected Mr-NPC2a:GFP in cell lysates though not in liquid culture, indicating that Mr-NPC2a is an intracellular protein as reported for other NPC2s (Fig. 5*C*) [28]. There are other reports of failure in detecting GFP signal in fungal transformants expressing a hybrid gene, and the reason for the failure is not clear [31].

### Mr-NPC2a is essential for membrane integrity of hyphal bodies

**Figure 6:**
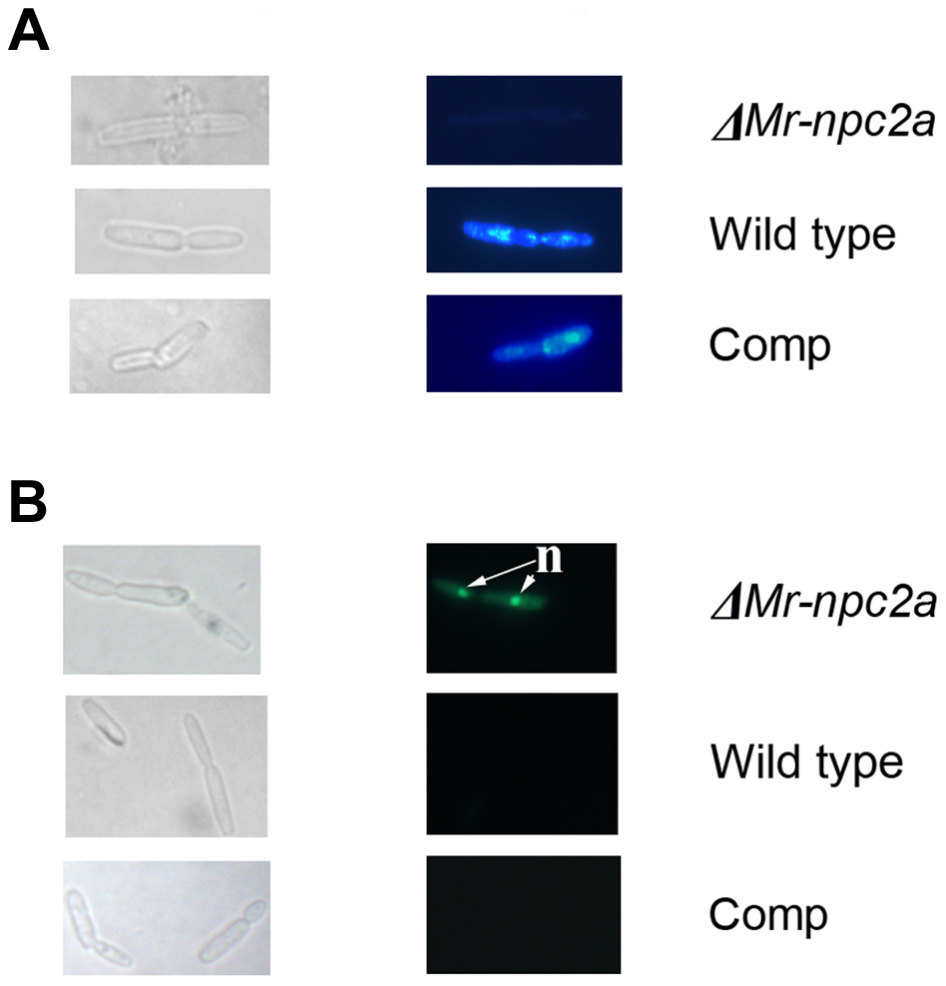
Filipin and sytox green staining of *M. robertsii* hyphal bodies collected from the hemolymph of living insects. Left panels: differential interference contrast images of hyphal bodies; Right panels: fluorescence microscopy of the same hyphal bodies shown in the left panels. (*A*) Filipin staining of sterols in the cell membrane, (*B*) Sytox staining. *ΔMr-npc2a*: the mutant with the ORF of *Mr-npc2a* deleted; WT: the wild type strain of *M. robertsii*; Comp: the complemented *ΔMr-npc2a* with a genomic clone of *Mr-npc2a*; n = nuclei.

*M. robertsii* rapidly proliferates as hyphal bodies in host hemolymph. Having confirmed that Mr-NPC2a is a cholesterol carrier and specifically expressed in hemolymph, we investigated its role in hyphal body formation. Flipin staining confirmed a large reduction in sterol content of *ΔMr-npc2a* hyphal bodies relative to those of wild type and the complemented *ΔMr-npc2a* (Fig. 6*A*), indicating a role in maintaining sterols as the fungus proliferates. When cultured in a complex nutrient medium (PDA), *ΔMr-npc2a*, the wild-type and the complemented *ΔMr-npc2a* cells had similar levels of cell membrane sterols (Fig. S4).

The nucleic acid stain Sytox is unable to cross intact cell membranes and therefore only stains cells with poor membrane integrity [32]. We found that 7±2.7% of wild type hyphal bodies, 8±3.1% of the complemented *ΔMr-npc2a* and 55±6.5% of *ΔMr-npc2a* hyphal bodies were permeable to Sytox (Fig. 6*B*), confirming that the provision of sterols by Mr-NPC2a has an important role in maintaining the membrane integrity of hyphal bodies. This result is consistent with *ΔMr-npc2a* producing fewer hyphal bodies in hemolymph than the wild type strain.

### Expression of Mr-NPC2a increased the virulence of the entomopathogenic fungus *Beauveria bassiana*

**Figure 7:**
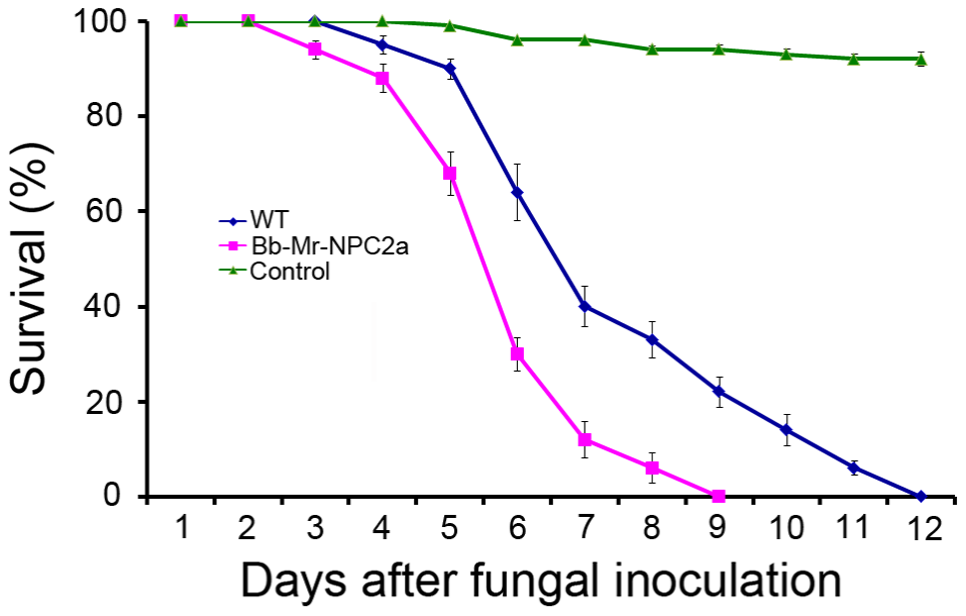
Kinetics of insect survivorship in bioassays with *B. bassiana*. Wax worm larvae (*G. mellonella*) were inoculated with *B. bassiana* conidial suspensions (1×10^7^ spores/mL). Blue: the wild type *B. bassiana*; Purple: a *B. bassiana* transformant (Bb-Mr-NPC2a) expressing Mr-NPC2a; Green: control insects that were treated with 0.05% Tween-80.

The genome of the well-studied insect-pathogenic fungus *Beauveria bassiana* (Genbank accession number: ADAH00000000) was searched for Mr-NPC2a homologs using BLASTP and TBLASTN (e-value cutoff 1e^−05^), but no significant hits were identified suggesting that this fungus lacks a Mr-NPC2a homolog. So, we decided to test whether transfer of Mr-NPC2a into *B. bassiana* can enhance its pathogenicity. Expression of Mr-NPC2a did not alter the growth, conidiation and germination of *B. bassiana*. Compared to the wild type strain, the LT_50_ (time taken to kill 50% of insects) of a transformant expressing Mr-NPC2a (Bb-Mr-NPC2a) was reduced by 22% (Fig. 7). Filipin staining did not detect obvious differences in sterol in the hyphal bodies of *B. bassiana* in hemolymph, but Sytox staining showed that the transformant expressing Mr-NPC2a had significantly fewer hyphal bodies (5±1.2%) with impaired cell membranes than did the wild type strain (10±1.8%).

## Discussion

The virulence of most fungal pathogens of plants and animals is a quantitative attribute, and thus many different genes and strategies are used simultaneously to cause disease [33]. Through evolution, fungi have acquired new virulence mechanisms, and increases in pathogen virulence have likely therefore evolved in a stepwise manner [34]. The important role of HGT in the evolution of fungal virulence is being illuminated by comparative genomics, but in only a few cases has the importance of the transferred genes been functionally demonstrated with gene knockouts, and no reports describe functional characterization of genes transferred from an animal to a eukaryotic pathogen. Lack of functional characterization is a major bottleneck in understanding the contribution of HGT to pathogen virulence. In this study, we describe how the insect pathogenic fungus *Metarhizium* may have acquired a sterol carrier gene *Mr-npc2a* from its insect hosts by HGT. This sterol carrier is involved in maintaining cell membrane sterols and thus membrane integrity of hyphal bodies in insect hemolymph. It thus helps *Metarhizium* adapt to the hemolymph and facilitates colonization of the host. A feature of *Mr-npc2a* is its specificity for the hemolymph of living insects. The promoter will thus be useful for driving expression of insecticidal proteins, and for manipulating gene expression in hemolymph, via antisense or overexpression constructs, to test whether candidate proteins play critical roles.

Ergosterol is an important constituent of fungal membrane lipids, similar to animal cholesterol, and modulates the fluidity, permeability and thickness of the membrane [35]. Under aerobic conditions, fungi synthesize ergosterol *de novo* and this process is oxygen dependent [36]. Under anaerobic conditions, ergosterol biosynthesis is suppressed and fungi need to take up sterols from the environment [37]. *Metarhizium* infects insects by direct penetration of the cuticle and entry into the haemocoel where the fungus kills insects with a combination of toxins and invasive growth by hyphal bodies [18]. However, the insect haemocoel is a hypoxic or even oxygen free environment, necessitating *M. robertsii* takes up sterols for the rapid proliferation of hyphal bodies. The insect hemolymph is the only environment where the fungal hyphal bodies can acquire sterols, and cholesterol is most likely to be exploited because it is the dominant insect sterol [38]. The bulk membrane function of the fungal ergosterol can be provided by structurally related sterols, including cholesterol [39]. Fungi can convert cholesterol into ergosterol or incorporate cholesterol directly into plasma membranes [28], [40].

Cholesterol is insoluble and insects use low density lipoproteins (LDL) to transport cholesterol through hemolymph to target tissues where they bind to LDL receptors. *Metarhizium* has a variety of genes with predicted functions that mimic those in insects including an LDL receptor (MAA_08841, Genbank accession no.: EFY95697) and close homologs of the *S. cerevisiae* ATP-binding cassette transporters AUS1 [*M. robertsii's* MAA_10338 (Genbank accession no.: EFY94212) and *M. acridum's* MAC_09600 (Genbank accession no.: EFY94889)] and PDR11 [*M. robertsii's* MAA_01883 (Genbank accession no.: EFY02301) and *M. acridum's* MAC_07971(Genbank accession no.: EFY96474)], required for cholesterol uptake in yeast [41].

The mechanisms of intracellular cholesterol transport are largely unknown, however, in mammals, cholesterol is exported to organelles by an NPC team that comprises an NPC1 (a lysosomal membrane protein) and NPC2 (a soluble protein in the lysosomal lumen). These mechanisms seem to be conserved in yeast and other fungi [28], [42]. *M. robertsii* has 1 NPC1-like protein (MAA_02001, Genbank accession no.: EFZ02419) as well as the 3 NPC2 proteins described in this study. Mr-NPC2b and Mr-NPC2c are typical fungal NPC2-like proteins, and their homolog in yeast (yNPC2p) is involved in ergosterol homeostasis [28]. The insect-like Mr-NPC2a is specifically expressed in insect hemolymph and is involved in maintaining cell membrane integrity only after the fungus enters the haemocoel. Although the disruption of *Mr-npc2a* greatly reduced the number of hyphal bodies in the hemolymph of living insects, i.e. suppressed growth, *ΔMr-npc2a* was still able to kill insects, suggesting that unidentified components can partially substitute for Mr-NPC2a when *M. robertsii* is in the hemolymph. The competition for cholesterol from *Metarhizium* might reduce the amount of sterols available to insects, which could alter the development of insects because insects cannot synthesize sterols and thus have dietary requirement for sterols. However, we did not see significant differences in development (pupation) between *G. mellonella* larva infected by *ΔMr-npc2a* and the wild type strain.

The existence of insect NPC2a homologs both in *Metarhizium* and the ergot fungus *C. purpurea* raises the question: when and how did these fungi acquire NPC2a from insects? There are four possible explanations. First, ancestral fungi had NPC2a proteins that were homologs of insect NPC2 proteins, but during evolution they were only retained by *Metarhizium* spp. and *C. purpurea*. This is unlikely as it would require independent loss of *npc2a* genes in all other fungal lineages. Secondly, *Metarhizium* and *C. purpurea* belong to the family Clavicipitaceae, and it is possible HGT occurred into the common ancestor of *Metarhizium* and *C. purpurea*. But microsynteny of sequence around *C. purpurea npc2a* is conserved with many fungi including *Metarhizium*. spp, whereas the microsynteny of the sequence around *Metarhizium npc2a* (*Mr-npc2a*) showed no conservation with other fungi including *C. purpurea*. So, if the ancestor of *Metarhizium* and *C. purpurea* acquired *npc2a* gene from an insect, this *npc2a* gene had moved from its ancient position to its current area during speciation of *Metarhizium. spp*. There is no evidence for *Metarhizium npc2a* (*Mr-npc2a*) having ever been within a transposon so genome rearrangement rather than transposition would be required for this move. Around *Mr-npc2a* (MAA_03817), only the gene MAA_03815 (Genbank accession no.: EFZ01219) is possibly involved in sterol metabolism which encodes C-4 methylsterol oxidase-like protein (Table S5), but *M. acridum*'s C-4 methylsterol oxidase-like gene (MAC_09281, Genbank accession no.: EFY84691) does not cluster with its *npc2a* gene (MAC_00943 Genbank accession no.: EFY93160). Therefore, no obvious evolutionary force is seen that selected the genome rearrangement in *Metarhizium* genus. Thirdly, ancestors of *C. purpurea* and *Metarhizium* spp. may have independently acquired *npc2a* gene from ancestral insects. It is conceptually easy to understand an insect pathogenic fungus acquiring a gene from its host by HGT. In nature, *C. purpurea* also has physical contact with insects, as it produces ergot honeydew that attracts insects and these spread its conidia [25]. However, the gene adjacent to *M. robertsii npc2a* (*Mr-npc2a*) and that to *C. purpurea npc2a* are homologs with related genes in other fungi but not in insects. It is improbable that insect *npc2a* genes independently inserted beside the same gene in *M. robertsii* and *C. purpurea*. The final possibility is that an ancestor of *C. purpurea* or *Metarhizium* spp. acquired *npc2a* which was inserted beside the gene MAA_03818 in *Metarhizium* to become MAA_03817 (*M. robertsii npc2a*) or beside the gene CPUR_07480 in *C. purpurea* to become CPUR_07479 (*C. purpurea npc2a*). *C. purpurea* then obtained the two genes (the genes MAA_03818 and MAA_03817) from *Metarhizium* or vice versa. Ecological niche overlap allows HGT of gene clusters between fungi [8], making this the most likely possibility. The genetic distances between the generalist *M. robertsii* (can kill many kinds of insects) and the specialist *M. acridum* (specific for grasshoppers) is greater than that between most other species in the *Metarhizium* genus [43]. Since the ancestor of *Metarhizium* may have acquired *npc2a* gene from an insect before speciation and both genetically distant *M. robertsii* and *M. acridum* retain this gene, it is expected that most of other *Metarhizium* species still have this gene. Therefore, based on currently available information, more *Metarhizium* than *Claviceps* species have *npc2a* genes, so it is more likely that *C. purpurea* was the recipient. However, more *Claviceps* genomes will be needed to confirm the direction of HGT.

## Materials and Methods

### Fungal and bacterial strains

*M. robertsii* ARSEF2575 and *B. bassiana* ARSEF252 were cultured as previously described [18], [44]. *Escherichia coli* DH5α was used for plasmid construction. *A. tumefaciens* AGL1 was used for fungal transformation as described [45].

### Gene cloning and disruption

The flanking sequences of T-DNA in mutants generated by the insertion of T-DNA into the genome of *M. robertsii* were cloned by Y-shaped adaptor-dependent extension as described [46]. The primers employed in this study and their usages are given in Table S6. PCR products were cloned into pGEM-T Easy (Promega, USA) for sequencing.

To construct the *Mr-npc2a* disruption plasmid, the 5′-end and 3′-end of *Mr-npc2a* were cloned by PCR and inserted into the *Xba*I and *Spe*I sites, respectively, of the plasmid pFBARGFP [18] to form the disruption plasmid pFBARGFP-dMr-npc2a. The disruption mutant (*ΔMr-npc2a*) was obtained utilizing *A. tumefaciens*-mediated fungal transformation. To complement *ΔMr-npc2a*, the genomic sequence of *Mr-npc2a* was cloned, inserted into the *Xba*I site of pSURGFP [43], and transformed into *ΔMr-npc2a* as described [47].

### Phylogenetic analyses and tree topology tests

*M. robertsii* Mr-NPC2a protein sequence was used as the query for BLASTP searches against the NCBI non-redundant database (e-value cutoff 1e^−05^) to identify and retrieve its homologs. The selected homologous proteins of representative taxa were aligned using MUSCLE 3.7 [48], with a maximum number of iterations of 16. Poorly aligned positions and divergent regions of the alignment were removed using GBlocks0.91b [49], with half the gapped positions allowed, the minimum number of sequences for a conserved and a flank position set to 50% of the number of taxa plus one, the maximum of contiguous nonconserved positions set to 16, and the minimum length of a block set to 4. Phylogenetic analyses were performed with three approaches: maximum likelihood (ML), bayesian inference (BI) and distance-based neighbor-joining (NJ). ML phylogenies were constructed by PhyML 3.1 [50] using the best-fit evolutionary model as suggested by ProtTest 3.2 [51], in which case, a discrete gamma-distribution model with four rate-categories plus invariant positions was used. The gamma parameter and proportion of invariant sites were estimated from the data. Branch support values were obtained by 100 bootstrap pseudo-replicates. Bayesian reconstructions were performed with MrBayes 3.1.2 [52] under the WAG+Γ4+I model of amino acid substitution. The Markov chain Monte Carlo searches were run for 1,000,000 generations, sampling the Markov chains every 1000 generations; the first 250 trees were discarded as ‘burn-in’. In addition, we also employed distance-based neighbor-joining (NJ) trees, which were constructed by using the program Neighbor in MEGA5 [53]. Bootstrap support values were obtained by generating 1,000 pseudo-replicates.

We statistically tested the significance of the topology of the obtained tree (Fig. 2) by phylogenetic analysis in comparison with alternative trees in which fungal NPC2a proteins were constrained to the vertebrate clade to form monophyletic groups using the program CONSEL [24]. The value of site-wise likelihood of every constrained topology was calculated by PhyML [50].

### Sterol binding assay of Mr-NPC2a

The DNA fragment encoding Mr-NPC2a (signal peptide excluded) was cloned with RT-PCR using total RNA extracted from hyphal bodies collected from the hemolymph of living insects as described below. The sequences of the primers are presented in Table S6. PCR product was purified and cloned into pGEM-T vector (Promega, Madison, WI, USA) for sequence verification. The DNA fragment was then subcloned into *Eco*RI/*Not*I restriction sites in the expression vector pET32a (EMD Millipore Corporation, Billerica, MA, USA) to form the pET32a-MrNPC2a plasmid that was then transformed into *E. coli* strain BL21 (DE3) (EMD Millipore Corporation, Billerica, MA, USA). Isopropyl-1-thio-β-D-galactopyranosid (1 mM) was used to induce recombinant protein expression. Recombinant Mr-NPC2a was purified with HisTALON Superflow Cartridges (Clontech, Mountain View, CA, USA), concentrated and dialyzed with Tris buffer (50 mM Tris-HCl, 50 mM NaCl, pH 8.0) at 4°C using Amicon Ultra centrifugal filter unit (EMD Millipore Corporation, Billerica, MA, USA). Control protein was expressed and purified from BL21 (DE3) cells transformed with pET32a empty vector with the same methods described above.

The ligand-binding assay was performed as described with some modifications [27]. Since the *D. melangaster* NPC2a can bind to bacterial Lipid A and cholesterol [27], both of them were used as ligands to compare binding specificity of Mr-NPC2a with that of the insect NPC2a. Ergosterol was also tested to look at the binding ability of Mr-NPC2a to the fungal sterol. Lipid A monophosphoryl from *E. coli* F583 (Rd mutant) and cholesterol were purchased from Sigma Aldrich (MO, USA), and ergosterol was from Santa Cruz Biotechnology (Texas, USA). Wells of flat-bottom 96-well plates (BD falcon, NJ, USA) were coated with 2 or 10 µg/well of each ligand. Control wells with no ligand were coated with the same amount of chloroform/water. The plates were then air dried overnight at room temperature, heated at 60°C for 30 min and then blocked with 200 µL/well of 1 mg/mL BSA in Tris buffer (50 mM Tris-HCl, 50 mM NaCl, pH 8.0) at 37°C for 2 h. Wells were washed with the Tris buffer (four times, each for 5 min) and 500 nM of purified Mr-NPC2a fusion protein or vector protein diluted in binding buffer (the Tris buffer with 0.1 mg/mL BSA) were added to each well of the coated plates (50 µL/well) and incubated at room temperature for 3 h. Plates were then washed and mouse monoclonal anti-polyHistidine antibody was added (100 µL/well) and incubated at 37°C for 2 h. Plates were washed again and alkaline phosphatase-conjugated goat anti-mouse IgG (Promega, 1∶2000 dilution in binding buffer) was added (100 µL/well) and incubated at 37°C for 2 h. Alkaline phosphatase substrate liquid (Sigma Aldrich MO, USA) was then added to each wells and incubated for 15 min at room temperature. Plates were shaken for 15 s and the absorbance at 405 nm of each well was determined using a plate reader (FilterMax F3, Molecular Devices, CA, USA). The absorbance from control wells was subtracted and specific binding was calculated by subtracting absorbance from vector protein from the total binding of NPC2 protein. Binding assays were repeated 3 times with 3 repeated wells.

### Expression pattern of *Mr-npc2a*

The expression pattern of *Mr-npc2a* was investigated by RT-PCR and by following GFP fluorescence in transformants expressing GFP driven by the promoter region (2,203 bp) of *Mr-npc2a*. The promoter region of the *Mr-npc2a* and the ORF of *egfp* were both cloned by PCR using primers described in Table S6. The resultant *egfp* product was digested with *Eco*RV and *Xho*I, and inserted into the corresponding sites of pBARGPE1 [30] to form pGFP. The *Mr-npc2a* promoter was digested with *Eco*RI and *Sma*I, and inserted into *Eco*RI and *Eco*RV of pGFP to form PMr-NPC2a:GFP. The *egfp* cassette was then mobilized into Ppk2-bar [21] to form pPMr-NPC2a:GFP. This Ti plasmid was then transformed into the wild type *M. robertsii* mediated by *A. tumefaciens* [45]. For RT-PCR analysis, the *gpd* gene was used as a reference gene [54]. Primers for *Mr-npc2a* and *gpd* are presented in Table S6. Total RNA was isolated using the Plant RNeasy Kit (Qiagen USA). First-strand cDNA was synthesized using Revert Aid First Strand cDNA Synthesis Kit (Fermentas, USA). PCR was conducted using DreamTaq DNA polymerase (Thermo Scientific, USA).

Expression of *Mr-npc2a* was examined during saprophytic growth, infection and in several possible inducing conditions (Table S4). For saprophytic growth, the fungus was grown on PDA, aerial hyphae, conidiating mycelium and conidia were sampled as previously described [23] for GFP observation or RNA preparation. Blastospores were obtained by growing the fungus in nutrition rich medium SDB for 10 d and in the *in vitro* prepared hemolymph for 2 d. GFP fluorescence in blastospores and hyphae was examined directly under microscopy. Blastospores and hyphae were combined for RNA preparation.

The expression of *Mr-npc2a* during infection was examined in appressoria on insect cuticles (the hindwings of *L. migratoria* locusts) and hyphal bodies in the hemolymph of living insects. Appressoria were induced on locust hindwings as previously described [55]. To obtain hyphal bodies in insect hemolymph, 1×10^5^ conidia were injected into a 5^th^ instar *M. sexta* larvae. After 20 h at 27°C, the insect was bled and GFP signal in the fungus in the hemolymph was checked by microscopy. The hemolymph with insect hemocytes and fungal hyphal bodies was subjected to RNA preparation. RNA of the hemolymph of healthy insects was prepared as a control for RT-PCR analysis.

In order to test the effect of sterols on the *Mr-npc2a* expression, the wild type strain (for RT-PCR analysis) or the transformant with PMr-npc2a:GFP (for GFP fluorescence observation) were grown in SDB for 24 h. The mycelium were then filtered, washed 3 times with sterile water and cultured in the minimal medium M100 [21] supplemented with the insect steroid hormone 20-Hydroxyecdysone (Sigma, USA), cholesterol (Sigma, USA) or ergosterol (Sigma, USA) (Table S4). To detect the effect of stresses on *Mr-npc2a* expression, the mycelium from the 24 h culture in SDB were cultured in the minimal medium M100 supplemented with different agents to generate stresses described in Table S4. For all treatments, GFP fluorescence in mycelium was examined and RNA was also prepared for RT-PCR.

### Investigating localization of Mr-NPC2a

To investigate the localization of Mr-NPC2a in *M. robertsii*, we constructed the fusion protein Mr-NPC2a: GFP. The coding sequence of Mr-NPC2a (stop codon was excluded) was cloned by PCR, and inserted into the *Eco*RV site of the plasmid pGFP described above, resulting in pMr-NPC2a:GFP. Since Mr-NPC2a is only expressed in hyphal bodies produced in the hemolymph of living insects, it is hard to determine the localization of this protein when its native promoter is used to drive the expression of NPC2a:GFP. Therefore, the expression of the fusion gene was driven by the constitutive promoter *Pgpd* from *A. nidulans* in pGFP [30]. The *Mr-NPC2a:GFP* cassette was removed from pMr-NPC2a:GFP and inserted into Ppk2-bar to produce Ppk2-gpd:Mr-NPC2a:GFP that was then transformed into wild type *M. robertsii* mediated by *A. tumefaciens* to produce transformant Mr-NPC2a:GFP.

GFP observation and Western blot analysis were used to test the localization of Mr-NPC2a:GFP. The transformant Mr-NPC2a:GFP was inoculated in SDB broth (10^6^ spores/mL) and grown for 36 h at 27°C with constant shaking (200 rpm). Mycelium was collected by filtration and ground into powder for protein preparation. The protein in the filtrant was precipitated with 80% (NH_4_)_2_SO_4_. The salt was then removed by centrifugation with Microcon filter with a 3 kDa cutoff (Millipore, USA). Total protein (15 µg) was subjected for Western blot analysis. Rabbit anti-GFP antibody (Proteintech Group Inc. USA) was used for Western blot analysis.

### Expression of Mr-NPC2a in *B. bassiana*

The ORF of *Mr-npc2a* was cloned by PCR and inserted into *Bam*HI and *Eco*RV sites downstream of the *Pgpd* promoter in the plasmid pBARGPE1 [30]. The *Mr-npc2a* expression cassette was then moved into pFBARGFP to form the expression plasmid pMr-NPC2a that was transformed into *B. bassiana* using *A. tumefaciens*.

### Sytox staining of hyphal bodies collected from the hemolymph of living insects

Sytox Green nucleic acid staining is widely used to assess the integrity of eukaryotic and prokaryotic cell membranes [32]. Sytox Green can only enter cells if their cell membrane integrity is compromised, in which case it stains nucleic acids, resulting in a green fluorescence emission (absorption and emission maxima at 502 and 523 nm, respectively). To prepare hyphal bodies for Sytox staining, 50 µL of conidial suspension (10^7^ conidia/mL) was injected into a fifth instar larva of *M. sexta*. After 20 h incubation at 27°C, the insect was bled and the hemolymph was collected into a 1.5 ml centrifuge tube that was then placed on ice. The hemolymph (100 µL) was then quickly mixed with 5 µL of sytox staining solution (10 µM) by pipetting up and down. The mixture was then immediately loaded on a glass slide and covered with a cover slip. This set was subsequently placed on wet paper towel in a small container and incubated at room temperature for 10 min. Stained nuclei were observed under microscopy.

### Flipin staining

Hyphal bodies were prepared from the hemolymph of *M. sexta* as described above and stained with 5 µM Filipin (Sigma Aldrich, MO, USA) in ACES buffer as described [28].

### Quantification of hyphal bodies in insect hemolymph

A fungal spore suspension (1×10^7^ spores/mL) was topically applied on the wax worm larvae (*G. mellonella*). At 12 h intervals after inoculation, ten larvae per treatment were surface sterilized with 1% bleach and individually bled as described [56]. Five microliter aliquots of blood from each larva were mixed with 95 µL of sterile water and spread onto *Metarhizium* selective medium for colony counts [56]. The number of colony forming units resulting from unicellular hyphal bodies (blastospores) or multicellular hyphal bodies is used to show the number of hyphal bodies.

### Bioassay

Fungal virulence was assayed against wax worm (*G.mellonella*) as previously described [56]. The worms were maintained as manufacture's instruction (Pet Solutions, OH USA). The SPSS program was used to calculate the LT_50_ for each strain. There were 40 insects for each strain and the experiment was repeated 3 times.

## Supporting Information

Figure S1 Diagram of the *Mr-npc2a* alleles in *ΔMr-npc2a*, the T-DNA insertion mutant M298 and the wild type strain. Wild type: the native *Mr-npc2a* gene and its promoter and termination regions in *M. robertsii* genome; M298: a T-DNA insertion mutant with *Mr-npc2a* gene disrupted. The T-DNA bordered by LB (left border) and RB (right border) is inserted inside the open reading frame (ORF) of *Mr-npc2a*, and a 302 bp long DNA fragment is deleted. Primers L1/L2 and R1/R2 are used to clone genomic DNA fragments adjacent to LB and RB, respectively. *ΔMr-npc2a*: the gene disruption mutant based on homologous recombination. The open reading frame of *Mr-npc2a* is replaced by the herbicide resistance gene *bar* cassette (Bar).
(TIF)

Figure S2 The disruption of *Mr-npc2a* in *M. robertsii*. (***A***) Left panel: the disruption plasmid of *Mr-npc2a* (bottom) and the relative position of the *Mr-npc2a* in the wild type strain (top). Based on homologous recombination, the ORF of Mr-npc2a in *M. robertsii* genome is replaced by herbicide resistance gene cassette. Right panel: screening of mutants with *Mr-npc2a* ORF deleted is based on GFP observation and herbicide resistance. Top: mutants are resistant to the herbicide with no GFP signal, showing that *Mr-npc2a* ORF is deleted without T-DNA inserted into other parts of the genome. Middle: transformants are resistant to the herbicide with GFP signal. In these transformants, T-DNA is randomly inserted in the genome and *Mr-npc2a* t is not disrupted. Bottom: transformants are resistant to the herbicide with GFP signal. In these transformants, *Mr-npc2a* ORF is deleted, but T-DNA inserts into other parts in the genome which could disrupts other genes. Transformants with GFP signal (middle and bottom panels) will be discarded. (***B***) Further confirmation of the deletion of *Mr-npc2a* ORF by PCR in the mutants with herbicides resistance and without GFP signal which were obtained from above screening. 1 and 2 are two mutants, and C is the wild type strain. Top panel: PCR conducted with the primers Bar-UP and CF2; Bottom panel: PCR conducted using primers CF1 and CF2. The positions of the primers are shown in the left panel of (*A*). The PCR data and the screening data [right panel in (A)] selected out the mutants where only *Mr-npc2a* ORF is deleted and no other genes are disrupted. (**C**) PCR confirmation of the complementation of *ΔMr-npc2a*. A genomic DNA fragment of *Mr-npc2a* including the promoter region, ORF and termination region was cloned by PCR using primers Mr-npc2a-5 and Mr-npc2a-3 (Table S6) and inserted into pPK2-SUR-gfp to form Ppk2-SUR-GFP-Mr-npc2a (Top panel) that was then transferred in to *ΔMr-npc2a*. Bottom panel: Confirmation of the complementation of *ΔMr-npc2a* by PCR using the primers F (Mr-npc2aORF-5) and R (Mr-npc2aORF-3) that were used to amplify the deleted region (ORF). 1 to 6: six different transformants with *ΔMr-npc2a* complemented; C: wild type strain; M: *ΔMr-npc2a*. The primers are described in Table S6.
(TIF)

Figure S3 Phylogeny of Mr-NPC2b and Mr-NPC2c and their homologs. The Bayesian inference tree is shown unrooted. Numbers at nodes represent Bayesian posterior probabilities. The scale bar corresponds to the estimated number of amino acid substitutions per site. This tree shows that the phylogenetic relationship between these proteins is consistent with previously established species phylogenies, demonstrating vertical inheritance.
(TIF)

Figure S4 Filipin staining of *M. robertsii* conidia collected from a PDA plate (Potato dextrose agar). Left panels: differential interference contrast images of conidia; Right panels: fluorescence due to Filipin staining of ergosterol in the cell membrane of the same conidia shown in the left panels. *ΔMr-npc2a*: A *M. robertsii* strain with *Mr-npc2a* deleted; Wild type: the wild type *M. robertsii* strain; Comp: the complemented *ΔMr-npc2a*.
(TIF)

Table S1 The number of hyphal bodies (hyphal bodies/mL) in the hemolymph of living wax worms.
(DOCX)

Table S2 GenBank accession numbers used for phylogenetic reconstruction in Figure 2.
(DOCX)

Table S3 Statistics of comparison of topologies of constrained trees with the tree obtained by phylogenetic analyses (non-constrained tree) (Fig. 2).
(DOCX)

Table S4 The induction of GFP expression in transformants with PMr-NPC2a:GFP.
(DOCX)

Table S5 Information about the genes around *Mr-npc2a* in *M. robertsii*.
(DOCX)

Table S6 Primers used in this study.
(DOCX)

## References

[1] Voyles J, Young S, Berger L, Campbell C, Voyles WF, et al (2009) Pathogenesis of chytridiomycosis, a cause of catastrophic amphibian declines. Science 326 5952: 582–585. 19900897 10.1126/science.1176765

[2] Lorch JM, Meteyer CU, Behr MJ, Boyles JG, Cryan PM, et al (2011) Experimental infection of bats with *Geomyces destructans* causes white-nose syndrome. Nature 480 7377: 376–378. 22031324 10.1038/nature10590

[3] Suh SO, Noda H, Blackwell M (2001) Insect symbiosis: derivation of yeast-like endosymbionts within an entomopathogenic filamentous lineage. Mol Biol Evol 18 6: 995–1000. 11371588 10.1093/oxfordjournals.molbev.a003901

[4] Ricklefs RE, Fallon SM (2002) Diversification and host switching in avian malaria parasites. Proc R Soc Lond B Biol Sci 269: 885–892. 10.1098/rspb.2001.1940 PMC1690983 12028770

[5] Archie EA, Luikart G, Ezenwa VO (2009) Infecting epidemiology with genetics: a new frontier in disease ecology. Trends Ecol Evol 24: 21–30. 19027985 10.1016/j.tree.2008.08.008

[6] Raffaele S, Farrer RA, Cano LM, Studholme DJ, MacLean D, et al (2010) Genome evolution following host jumps in the Irish potato famine pathogen lineage. Science 330: 1540–1543. 21148391 10.1126/science.1193070

[7] Gilbert C, Hernandez SS, Flores-Benabib J, Smith EN, Feschotte C (2012) Rampant horizontal transfer of SPIN transposons in squamate reptiles. Mol Biol Evol 29: 503–515. 21771716 10.1093/molbev/msr181 PMC3350315

[8] Slot JC, Rokas A (2011) Horizontal transfer of a large and highly toxic secondary metabolic gene cluster between fungi. Curr Biol 21 2: 134–139. 21194949 10.1016/j.cub.2010.12.020

[9] Friesen TL, Stukenbrock EH, Liu Z, Meinhardt S, Ling H, et al (2006) Emergence of a new disease as a result of interspecific virulence gene transfer. Nat Genet 38: 953–956. 16832356 10.1038/ng1839

[10] Richards TA, Leonard G, Soanes DM, Talbot NJ (2011) Gene transfer into the fungi. Fungal. Biol Rev 25: 98–110.

[11] Sun BF, Xiao JH, He S, Liu L, Murphy RW, et al (2013) Multiple interkingdom horizontal gene transfers in Pyrenophora and closely related species and their contributions to phytopathogenic lifestyles. PLoS One 8 3: e60029. 23555871 10.1371/journal.pone.0060029 PMC3612039

[12] Mower JP, Stefanovic S, Young GJ, Palmer JD (2004) Plant genetics: gene transfer from parasitic to host plants. Nature 432: 165–166. 10.1038/432165b 15538356

[13] Davis CC, Wurdack KJ (2004) Host-to-parasite gene transfer in flowering plants: phylogenetic evidence from Malpighiales. Science 305: 676–678. 15256617 10.1126/science.1100671

[14] Keeling PJ, Palmer JD (2008) Horizontal gene transfer in eukaryotic evolution. Nat Rev Genet 9: 605–618. 18591983 10.1038/nrg2386

[15] Selman M, Pombert JF, Solter L, Farinelli L, Weiss LM, et al (2011) Acquisition of an animal gene by microsporidian intracellular parasites. Curr Biol 21: 576–577. 10.1016/j.cub.2011.06.017 PMC3751409 21820617

[16] Bar DZ (2011) Evidence of massive horizontal gene transfer between humans and *Plasmodium vivax* . Nature precedings doi:10.1038/npre.2011.5690.1

[17] Roberts DW, St Leger RJ (2004) *Metarhizium* spp., cosmopolitan insect-pathogenic fungi: mycological aspects. Adv Appl Microbiol 54: 1–70. 15251275 10.1016/S0065-2164(04)54001-7

[18] Fang W, Scully LR, Zhang L, Pei Y, Bidochka MJ (2008) Implication of a regulator of G protein signalling (BbRGS1) in conidiation and conidial thermotolerance of the insect pathogenic fungus *Beauveria bassiana* . FEMS Microbiol Lett 279: 146–156. 18201190 10.1111/j.1574-6968.2007.00978.x

[19] Fang W, Azimzadeh P, St Leger RJ (2012) Strain improvement of fungal insecticides for controlling insect pests and vector-borne diseases. Curr Opin Microbiol 15: 232–238. 22245564 10.1016/j.mib.2011.12.012

[20] Behie SW, Zelisko PM, Bidochka MJ (2012) Endophytic insect-parasitic fungi translocate nitrogen directly from insects to plants. Science 336: 1576–1577. 22723421 10.1126/science.1222289

[21] Fang W, St Leger RJ (2010) Mrt, a gene unique to fungi, encodes an oligosaccharide transporter and facilitates rhizosphere competency in *Metarhizium robertsii* . Plant Physiol 154: 1549–1557. 20837701 10.1104/pp.110.163014 PMC2971628

[22] Xiao G, Ying SH, Zheng P, Wang ZL, Zhang S, et al (2012) Genomic perspectives on the evolution of fungal entomopathogenicity in *Beauveria bassiana* . Sci Rep 2: 483. 22761991 10.1038/srep00483 PMC3387728

[23] Fang W, Fernandes EK, Roberts DW, Bidochka MJ, St Leger RJ (2010) A laccase exclusively expressed by *Metarhizium anisopliae* during isotropic growth is involved in pigmentation, tolerance to abiotic stresses and virulence. Fungal Genet Biol 47: 602–607. 20382249 10.1016/j.fgb.2010.03.011

[24] Shimodaira H, Hasegawa M (2001) CONSEL: for assessing the confidence of phylogenetic tree selection. Bioinformatics 17 12: 1246–1247. 11751242 10.1093/bioinformatics/17.12.1246

[25] Butler MD, Alderman SC, Hammond PC, Berry RE (2001) Association of Insects and Ergot (*Claviceps purpurea*) in Kentucky Bluegrass Seed Production Fields. J Econ Entomol 94: 1471–1476. 11777051 10.1603/0022-0493-94.6.1471

[26] Danchin EG, Rosso MN, Vieira P, de Almeida-Engler J, Coutinho PM, et al (2010) Multiple lateral gene transfers and duplications have promoted plant parasitism ability in nematodes. Proc Natl Acad Sci USA 107 41: 17651–17656. 20876108 10.1073/pnas.1008486107 PMC2955110

[27] Shi XZ, Zhong X, Yu XQ (2012) *Drosophila melanogaster* NPC2 proteins bind bacterial cell wall components and may function in immune signal pathways. Insect Biochem Mol Biol 42: 545–556. 22580186 10.1016/j.ibmb.2012.04.002 PMC3358802

[28] Berger AC, Vanderford TH, Gernert KM, Nichols JW, Faundez V, et al (2005) *Saccharomyces cerevisiae* Npc2p is a functionally conserved homologue of the human Niemann-Pick disease type C 2 protein, hNPC2. Eukaryot Cell 4: 1851–1862. 16278452 10.1128/EC.4.11.1851-1862.2005 PMC1287848

[29] Wang C, St Leger RJ (2006) A collagenous protective coat enables *Metarhizium anisopliae* to evade insect immune responses. Proc Natl Acad Sci USA 103: 6647–6652. 16614065 10.1073/pnas.0601951103 PMC1458935

[30] McCluskey K (2003) The Fungal Genetics Stock Center: from molds to molecules. Adv Appl Microbiol 52: 245–262. 12964247 10.1016/s0065-2164(03)01010-4

[31] Chen P, Gao R, Chen S, Pu L, Li P, et al (2012) A pericentrin-related protein homolog in *Aspergillus nidulans* plays important roles in nucleus positioning and cell polarity by affecting microtubule organization. Eukaryot Cell 11: 1520–1530. 23087372 10.1128/EC.00203-12 PMC3536274

[32] Gerphagnon M, Latour D, Colombet J, Sime-Ngando T (2013) A double staining method using SYTOX green and calcofluor white for studying fungal parasites of phytoplankton. Appl Environ Microbiol 79: 3943–3951. 23603679 10.1128/AEM.00696-13 PMC3697587

[33] Horbach R, Navarro-Quesada AR, Knogge W, Deising HB (2011) When and how to kill a plant cell: infection strategies of plant pathogenic fungi. J Plant Physiol 168: 51–62. 20674079 10.1016/j.jplph.2010.06.014

[34] Gardiner DM, Kazan K, Manners JM (2013) Cross-kingdom gene transfer facilitates the evolution of virulence in fungal pathogens. Plant Sci 210: 151–158. 23849122 10.1016/j.plantsci.2013.06.002

[35] Zhang YQ, Gamarra S, Garcia-Effron G, Park S, Perlin DS, et al (2010) Requirement for ergosterol in V-ATPase function underlies antifungal activity of azole drugs. PLoS Pathog 6: e1000939. 20532216 10.1371/journal.ppat.1000939 PMC2880581

[36] Dupont S, Lemetais G, Ferreira T, Cayot P, Gervais P, et al (2012) Ergosterol biosynthesis: a fungal pathway for life on land? Evolution 66: 2961–2968. 22946816 10.1111/j.1558-5646.2012.01667.x

[37] Andreasen AA, Stier TJ (1953) Anaerobic nutrition of *Saccharomyces cerevisiae*. I. Ergosterol requirement for growth in a defined medium. J Cell Physiol 41: 23–36. 10.1002/jcp.1030410103 13034889

[38] Jing X, Grebenok RJ, Behmer ST (2013) Sterol/steroid metabolism and absorption in a generalist and specialist caterpillar: Effects of dietary sterol/steroid structure, mixture and ratio. Insect Biochem Mol Biol 43: 580–587. 23567589 10.1016/j.ibmb.2013.03.012

[39] Jacquier N, Schneiter R (2012) Mechanisms of sterol uptake and transport in yeast. J Steroid Biochem Mol Biol 129: 70–78. 21145395 10.1016/j.jsbmb.2010.11.014

[40] Xiong Q, Hassan SA, Wilson WK, Han XY, May GS, et al (2005) Cholesterol import by *Aspergillus fumigatus* and its influence on antifungal potency of sterol biosynthesis inhibitors. Antimicrob Agents Chemother 49: 518–524. 15673727 10.1128/AAC.49.2.518-524.2005 PMC547240

[41] Alimardani P, Regnacq M, Moreau-Vauzelle C, Ferreira T, Rossignol T, et al (2004) SUT1-promoted sterol uptake involves the ABC transporter Aus1 and the mannoprotein Dan1 whose synergistic action is sufficient for this process. Biochem J 381: 195–202. 15035656 10.1042/BJ20040297 PMC1133777

[42] Berger AC, Hanson PK, Wylie Nichols J, Corbett AH (2005) A yeast model system for functional analysis of the Niemann-Pick type C protein 1 homolog, Ncr1p. Traffic 6: 907–917. 16138904 10.1111/j.1600-0854.2005.00327.x

[43] Bischoff JF, Rehner SA, Humber RA (2009) A multilocus phylogeny of the *Metarhizium anisopliae* lineage. Mycologia 101: 512–530. 19623931 10.3852/07-202

[44] Fang W, Pei Y, Bidochka MJ (2007) A regulator of a G protein signaling (RGS) gene, cag8, from the insect-pathogenic fungus *Metarhizium anisopliae* is involved in conidiation, virulence and hydrophobin synthesis. Microbiology 153: 1017–1025. 17379711 10.1099/mic.0.2006/002105-0

[45] Fang W, Pei Y, Bidochka MJ (2006) Transformation of *Metarhizium anisopliae* mediated by *Agrobacterium tumefaciens* . Can J Microbiol 52: 623–626. 16917517 10.1139/w06-014

[46] Fang W, Leng B, Xiao Y, Jin K, Ma J, et al (2005) Cloning of *Beauveria bassiana* chitinase gene Bbchit1 and its application to improve fungal strain virulence. Appl Environ Microbiol 71: 363–370. 15640210 10.1128/AEM.71.1.363-370.2005 PMC544255

[47] Fang W, St Leger RJ (2012) Enhanced UV resistance and improved killing of malaria mosquitoes by photolyase transgenic entomopathogenic fungi. PLoS One 7: e43069. 22912789 10.1371/journal.pone.0043069 PMC3422317

[48] Edgar RC (2004) MUSCLE: multiple sequence alignment with high accuracy and high throughput. Nucleic Acids Res 32: 1792–1797. 15034147 10.1093/nar/gkh340 PMC390337

[49] Castresana J (2000) Selection of conserved blocks from multiple alignments for their use in phylogenetic analysis. Mol Biol Evol 17: 540–552. 10742046 10.1093/oxfordjournals.molbev.a026334

[50] Guindon S, Gascuel O (2003) A simple, fast, and accurate algorithm to estimate large phylogenies by maximum likelihood. Syst Biol 52: 696–704. 14530136 10.1080/10635150390235520

[51] Abascal F, Zardoya R, Posada D (2005) ProtTest: selection of best-fit models of protein evolution. Bioinformatics 21: 2104–2105. 15647292 10.1093/bioinformatics/bti263

[52] Huelsenbeck JP, Ronquist F (2001) MR BAYES: Bayesian inference of phylogenetic trees. Bioinformatics 17: 754–755. 11524383 10.1093/bioinformatics/17.8.754

[53] Tamura K, Peterson D, Peterson N, Stecher G, Nei M, et al (2011) MEGA5: Molecular evolutionary genetics analysis using maximum likelihood, evolutionary distance, and maximum parsimony methods. Mol Biol Evol 28: 2731–2739. 21546353 10.1093/molbev/msr121 PMC3203626

[54] Fang W, Bidochka MJ (2006) Expression of genes involved in germination, conidiogenesis and pathogenesis in *Metarhizium anisopliae* using quantitative real-time RT-PCR. Mycol Res 110: 1165–1171. 17010593 10.1016/j.mycres.2006.04.014

[55] Fang W, Pava-ripoll M, Wang S, St.Leger RJ (2009) Protein kinase A regulates production of virulence determinants by the entomopathogenic fungus, *Metarhizium anisopliae* . Fungal Genet Biol 46: 277–85. 19124083 10.1016/j.fgb.2008.12.001

[56] Fang W, Feng J, Fan Y, Zhang Y, Bidochka MJ, et al (2009) Expressing a fusion protein with protease and chitinase activities increases the virulence of the insect pathogen *Beauveria bassiana* . J Invertebr Pathol 102: 155–159. 19666027 10.1016/j.jip.2009.07.013

