# Supplementary figures and images for: Host-to-Pathogen Gene Transfer Facilitated Infection of Insects by a Pathogenic Fungus
Source: PLoS Pathog. 2014 Apr 10;10(4):e1004009. doi: 10.1371/journal.ppat.1004009 (PMC3983072; doi:10.1371/journal.ppat.1004009)

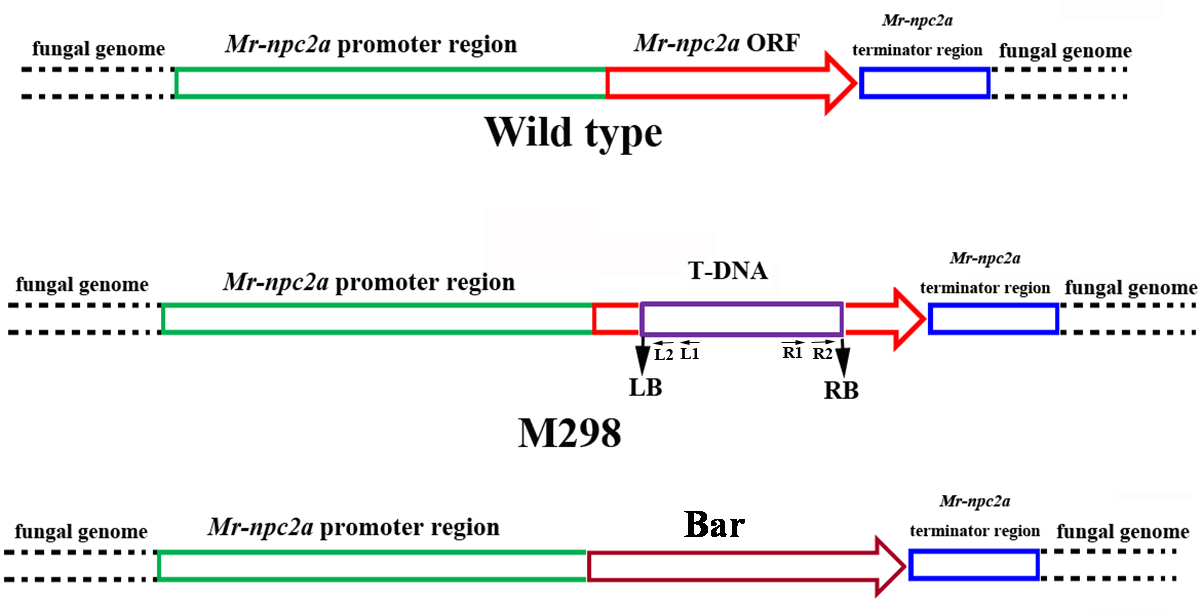

Supplement: Figure S1 — Diagram of the Mr-npc2a alleles in ΔMr-npc2a, the T-DNA insertion mutant M298 and the wild type strain. Wild type: the native Mr-npc2a gene and its promoter and termination regions in M. robertsii genome; M298: a T-DNA insertion mutant with Mr-npc2a gene disrupted. The T-DNA bordered by LB (left border) and RB (right border) is inserted inside the open reading frame (ORF) of Mr-npc2a, and a 302 bp long DNA fragment is deleted. Primers L1/L2 and R1/R2 are used to clone genomic DNA fragments adjacent to LB and RB, respectively. ΔMr-npc2a: the gene disruption mutant based on homologous recombination. The open reading frame of Mr-npc2a is replaced by the herbicide resistance gene bar cassette (Bar). (TIF) [file ppat.1004009.s001.tif]

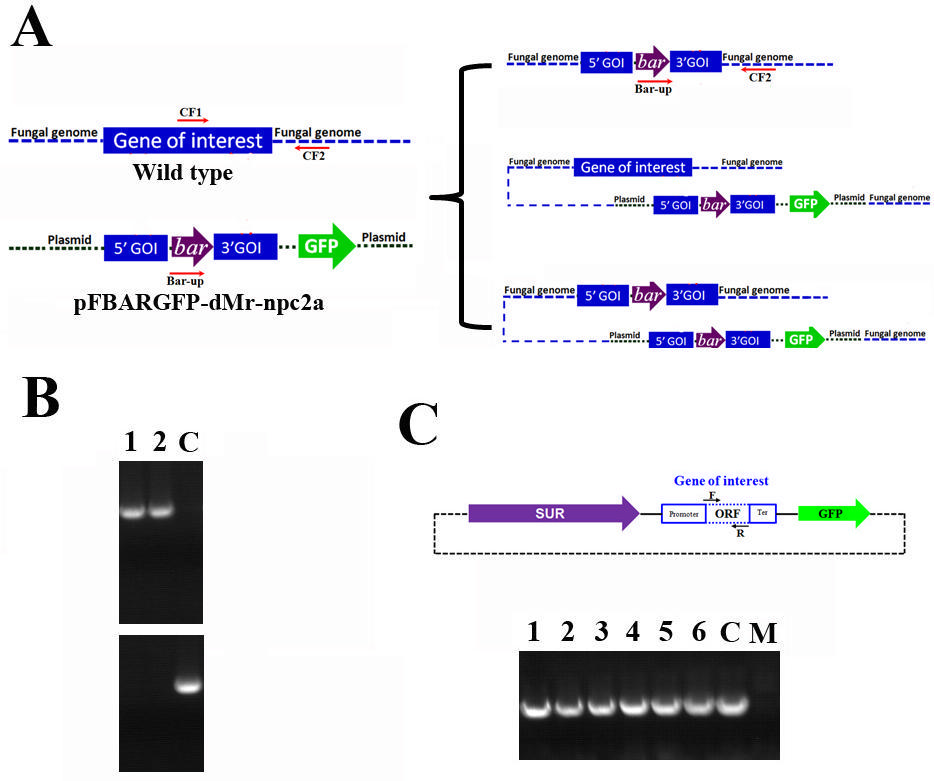

Supplement: Figure S2 — The disruption of Mr-npc2a in M. robertsii. (A) Left panel: the disruption plasmid of Mr-npc2a (bottom) and the relative position of the Mr-npc2a in the wild type strain (top). Based on homologous recombination, the ORF of Mr-npc2a in M. robertsii genome is replaced by herbicide resistance gene cassette. Right panel: screening of mutants with Mr-npc2a ORF deleted is based on GFP observation and herbicide resistance. Top: mutants are resistant to the herbicide with no GFP signal, showing that Mr-npc2a ORF is deleted without T-DNA inserted into other parts of the genome. Middle: transformants are resistant to the herbicide with GFP signal. In these transformants, T-DNA is randomly inserted in the genome and Mr-npc2a t is not disrupted. Bottom: transformants are resistant to the herbicide with GFP signal. In these transformants, Mr-npc2a ORF is deleted, but T-DNA inserts into other parts in the genome which could disrupts other genes. Transformants with GFP signal (middle and bottom panels) will be discarded. (B) Further confirmation of the deletion of Mr-npc2a ORF by PCR in the mutants with herbicides resistance and without GFP signal which were obtained from above screening. 1 and 2 are two mutants, and C is the wild type strain. Top panel: PCR conducted with the primers Bar-UP and CF2; Bottom panel: PCR conducted using primers CF1 and CF2. The positions of the primers are shown in the left panel of (A). The PCR data and the screening data [right panel in (A)] selected out the mutants where only Mr-npc2a ORF is deleted and no other genes are disrupted. (C) PCR confirmation of the complementation of ΔMr-npc2a. A genomic DNA fragment of Mr-npc2a including the promoter region, ORF and termination region was cloned by PCR using primers Mr-npc2a-5 and Mr-npc2a-3 (Table S6) and inserted into pPK2-SUR-gfp to form Ppk2-SUR-GFP-Mr-npc2a (Top panel) that was then transferred in to ΔMr-npc2a. Bottom panel: Confirmation of the complementation of ΔMr-npc2a by PCR using the primer [file ppat.1004009.s002.tif]

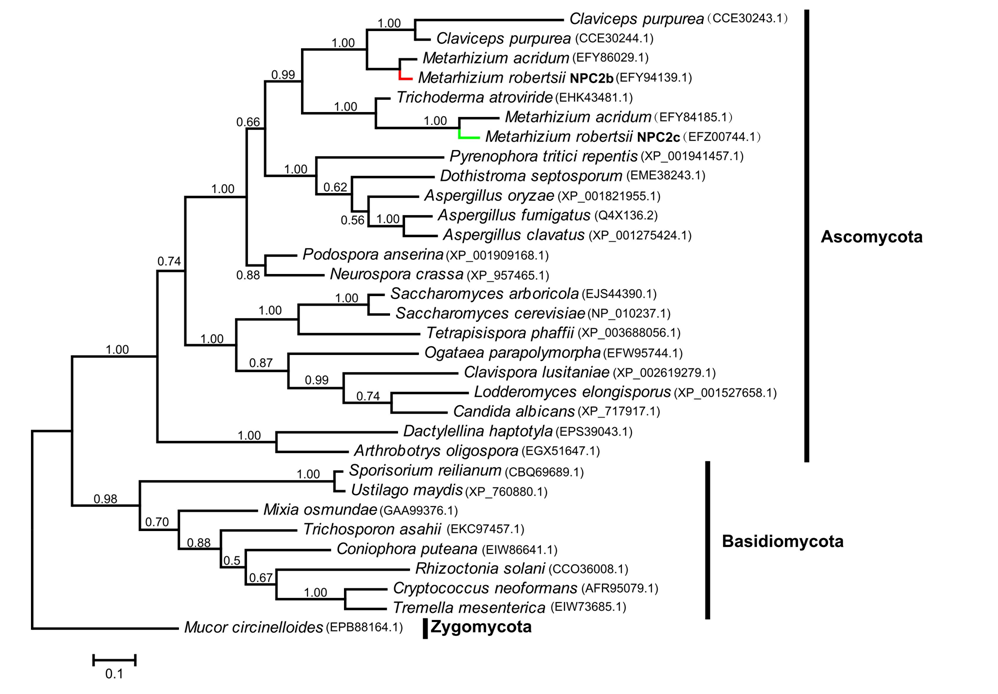

Supplement: Figure S3 — Phylogeny of Mr-NPC2b and Mr-NPC2c and their homologs. The Bayesian inference tree is shown unrooted. Numbers at nodes represent Bayesian posterior probabilities. The scale bar corresponds to the estimated number of amino acid substitutions per site. This tree shows that the phylogenetic relationship between these proteins is consistent with previously established species phylogenies, demonstrating vertical inheritance. (TIF) [file ppat.1004009.s003.tif]

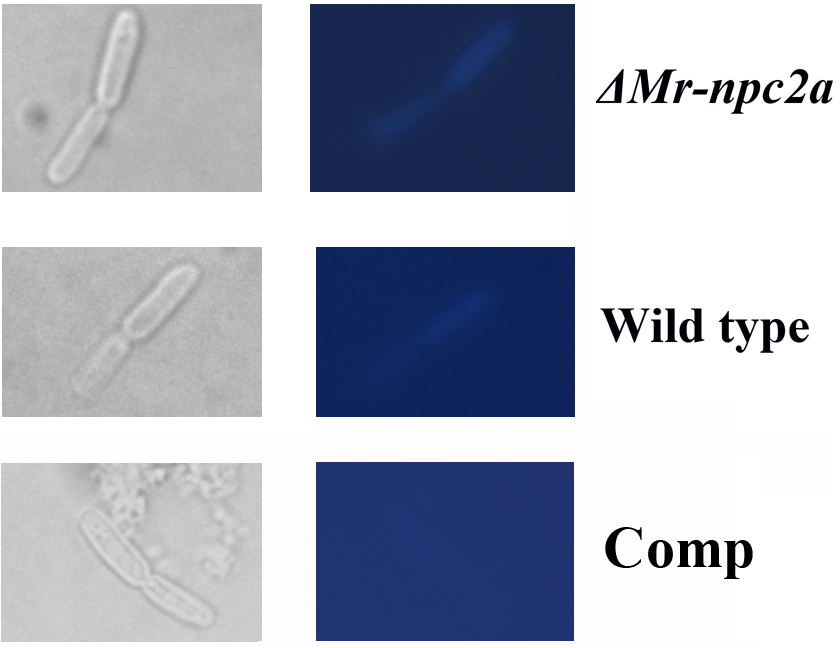

Supplement: Figure S4 — Filipin staining of M. robertsii conidia collected from a PDA plate (Potato dextrose agar). Left panels: differential interference contrast images of conidia; Right panels: fluorescence due to Filipin staining of ergosterol in the cell membrane of the same conidia shown in the left panels. ΔMr-npc2a: A M. robertsii strain with Mr-npc2a deleted; Wild type: the wild type M. robertsii strain; Comp: the complemented ΔMr-npc2a. (TIF) [file ppat.1004009.s004.tif]
