# Supplementary material for: Host-to-Pathogen Gene Transfer Facilitated Infection of Insects by a Pathogenic Fungus
Source: PLoS Pathog. 2014 Apr 10;10(4):e1004009. doi: 10.1371/journal.ppat.1004009 (PMC3983072; doi:10.1371/journal.ppat.1004009)
Supplement: Table S1 — The number of hyphal bodies (hyphal bodies/mL) in the hemolymph of living wax worms. (DOCX) [file ppat.1004009.s005.docx]

**Table S1**. The number of hyphal bodies (hyphal bodies/mL) in the hemolymph of living wax worms

| **Strains** | **36h** | | **48h** | | **60h** | |  |
| --- | --- | --- | --- | --- | --- | --- | --- |
| Wild type | | 36±9.3 | | 78±8.9 | | 198±15 | |
| *ΔMr-NPC2a* | | 20±3.8 | | 45±7.8 | | 105±16 | |
| M298 | | 22±3.2 | | 48±6.9 | | 112±21 | |
| The complemented *ΔMr-NPC2a* | | 38±9.5 | | 77±10 | | 201±21 | |
|  |  | |  | |  | |  |
