# Supplementary material for: Host-to-Pathogen Gene Transfer Facilitated Infection of Insects by a Pathogenic Fungus
Source: PLoS Pathog. 2014 Apr 10;10(4):e1004009. doi: 10.1371/journal.ppat.1004009 (PMC3983072; doi:10.1371/journal.ppat.1004009)
Supplement: Table S2 — GenBank accession numbers used for phylogenetic reconstruction in Figure 2. (DOCX) [file ppat.1004009.s006.docx]

**Table S2** GenBank accession numbers used for phylogenetic reconstruction in Figure 2.

| **Species Name** | **Accession** | **Group** |
| --- | --- | --- |
| *Dendroctonus ponderosae* | AEE61686.1 | Coleoptera |
| *Tribolium castaneum* | XP_975622.1 | Coleoptera |
| *Drosophila ananassae* | XP_001962129.1 | Diptera |
| *Drosophila melanogaster* | NP_608637.1 | Diptera |
| *Ceratitis capitata* | XP_004536369.1 | Diptera |
| *Culex quinquefasciatus* | XP_001847173.1 | Diptera |
| *Apis mellifera* | XP_624310.2 | Hymenoptera |
| *Bombus impatiens* | XP_003487321.1 | Hymenoptera |
| *Megachile rotundata* | XP_003700172.1 | Hymenoptera |
| *Acromyrmex echinatior* | EGI66092.1 | Hymenoptera |
| *Solenopsis invicta* | EFZ16828.1 | Hymenoptera |
| *Danaus plexippus* | EHJ65928.1 | Lepidoptera |
| *Papilio xuthus* | BAM18090.1 | Lepidoptera |
| *Nematostella vectensis* | XP_001622874.1 | Cnidaria(Hexacorallia) |
| *Claviceps purpurea* 20.1 | CCE33556.1 | Fungi(Clavicipitaceae) |
| *Metarhizium anisopliae* ARSEF 23 | EFZ01221.1 | Fungi(Clavicipitaceae) |
| *Metarhizium acridum* CQMa 102 | EFY93160.1 | Fungi(Clavicipitaceae) |
| *Anoplopoma fimbria* | ACQ58458.1 | Actinopterygii |
| *Salmo salar* | ACM08223.1 | Actinopterygii |
| *Danio rerio* | NP_775331.1 | Actinopterygii |
| *Gekko japonicus* | AAU14264.1 | Lepidosauria |
| *Anolis carolinensis* | XP_003214431.1 | Lepidosauria |
| *Gallus gallus* | NP_001026374.1 | Testudines + Archosauria group |
| *Mus musculus* | BAE31061.1 | Mammalia |
| *Homo sapiens* | NP_006423.1 | Mammalia |
| *Pan paniscus* | XP_003824213.1 | Mammalia |
| *Bos taurus* | NP_776343.1 | Mammalia |
| *Ovis aries* | XP_004010837.1 | Mammalia |
