# Supplementary material for: Host-to-Pathogen Gene Transfer Facilitated Infection of Insects by a Pathogenic Fungus
Source: PLoS Pathog. 2014 Apr 10;10(4):e1004009. doi: 10.1371/journal.ppat.1004009 (PMC3983072; doi:10.1371/journal.ppat.1004009)
Supplement: Table S3 — Statistics of comparison of topologies of constrained trees with the tree obtained by phylogenetic analyses (non-constrained tree) (Fig. 2). (DOCX) [file ppat.1004009.s007.docx]

**Table S3**. Statistics of comparison of topologies of constrained trees with the tree obtained by phylogenetic analyses (non-constrained tree) (Fig. 2)

| **Scenarios** | **obs** | **au** | **np** | **bp** | **pp** | **kh** | **sh** | **wkh** | **wsh** |
| --- | --- | --- | --- | --- | --- | --- | --- | --- | --- |
| **(((A,B),C),D)*** | **-2.5** | **0.83** | **0.797** | **0.795** | **0.761** | **0.81** | **0.811** | **0.81** | **0.811** |
| ((A,D),B,C) | 2.5 | 0.178 | 0.208 | 0.079 | 0.06 | 0.19 | 0.19 | 0.19 | 0.366 |
| (((A,D),B),C) | 2.5 | 0.178 | 0.208 | 0.079 | 0.06 | 0.19 | 0.19 | 0.19 | 0.366 |
| (((A,D),C),B) | 2.5 | 0.179 | 0.208 | 0.076 | 0.06 | 0.19 | 0.19 | 0.19 | 0.358 |
| ((A,D),(B,C)) | 2.5 | 0.176 | 0.209 | 0.051 | 0.06 | 0.19 | 0.19 | 0.19 | 0.387 |

***: the non-constrained tree presented in Fig. 2. Fungal clade, Culex clade, insect clade and vertebrata clade in Fig. 2 are designated as A, B, C and D, respectively. According to the non-constrained tree (Fig. 2), trees that do not support HGT between fungi and insects are the fungal NPC2a proteins cluster with vertebrate NPC2s, so selected and tested are only the constrained trees where the fungal NPC2a proteins (A) and vertebrate NPC2s (D) are put together to form monophyletic group. The value of site-wise likelihood of every constrained topology was calculated by PhyML. Hypothesis testing was implemented with CONSEL.
