# Supplementary material for: Host-to-Pathogen Gene Transfer Facilitated Infection of Insects by a Pathogenic Fungus
Source: PLoS Pathog. 2014 Apr 10;10(4):e1004009. doi: 10.1371/journal.ppat.1004009 (PMC3983072; doi:10.1371/journal.ppat.1004009)
Supplement: Table S4 — The induction of GFP expression in transformants with PMr-NPC2a:GFP. (DOCX) [file ppat.1004009.s008.docx]

**Table S4**. The induction of GFP expression in transformants with PMr-NPC2a:GFP

| **Inducer** | **GFP signal** | | **RT-PCR detection** |
| --- | --- | --- | --- |
| Sterols/lipids | | |  |
| Cholesterol (50 μg/ml) | - | | - |
| Ergosterol (50 μg/ml) | - | | - |
| 20-Hydroxyecdysone (5 μg/ml) | - | | - |
|  |  |  |  |
| Abiotic stresses |  | |  |
| oxidative stress |  | |  |
| H2O2 (0.03%) | - | | - |
| Diamide (3 μg ml^−1^) | - | | - |
| Methyl-viologen (22.5 μg ml^−1^). | - | | - |
| Osmotic stress |  | |  |
| 0.7M KCl | - | | - |
| 1.0 M KCl | - | | - |
| Hypoxia stress (1% O_2_, 5% CO_2_ and 94% N_2_) | - | | - |
|  |  | |  |
| *In vitro* prepared insect hemolymph* | - | | - |
|  |  |  |  |

*: many blastospores were produced in this culture and were also subjected to RNA preparation with hyphae.
