# Supplementary material for: Host-to-Pathogen Gene Transfer Facilitated Infection of Insects by a Pathogenic Fungus
Source: PLoS Pathog. 2014 Apr 10;10(4):e1004009. doi: 10.1371/journal.ppat.1004009 (PMC3983072; doi:10.1371/journal.ppat.1004009)
Supplement: Table S5 — Information about the genes around Mr-npc2a in M. robertsii. (DOCX) [file ppat.1004009.s009.docx]

**Table S5.** Information about the genes around *Mr-npc2a* in *M. robertsii*

| Genes | Functions (estimated by BLASTP) |
| --- | --- |
| MAA_03812  MAA_03813  MAA_03814  MAA_03815  MAA_03816  MAA_03817  MAA_03818  MAA_03819  MAA_03820  MAA_03821  MAA_03822 | Hypothetical protein  MFS transporter  Hypothetical protein  C4-methylsterol oxidase  Hypothetical proteins  NPC2 sterol carrier  Hypothetical protein  [glutamate decarboxylase](http://blast.ncbi.nlm.nih.gov/Blast.cgi#alnHdr_322709647)  Nitrogen response regulator  [serine/threonine-protein kinase sid2](http://blast.ncbi.nlm.nih.gov/Blast.cgi#alnHdr_322709649)  hypothetical protein |
