# Supplementary material for: Host-to-Pathogen Gene Transfer Facilitated Infection of Insects by a Pathogenic Fungus
Source: PLoS Pathog. 2014 Apr 10;10(4):e1004009. doi: 10.1371/journal.ppat.1004009 (PMC3983072; doi:10.1371/journal.ppat.1004009)
Supplement: Table S6 — Primers used in this study. (DOCX) [file ppat.1004009.s010.docx]

**Table S6**. Primers used in this study

| Primers | Sequence | Usage |
| --- | --- | --- |
| YAL  YAT-1  YAT-2  YAT-3  YADEE  L1  L2  R1  R2  DMr-npc2a5-1  DMr-npc25-2  DMr-npc2a3-1  DMr-npc2a3-2  DMr-npc2ACF-1  DMr-npc2ACF-2  PMr-npc2a-5  PMr-npc2a-3  Mr-npc2a-5  Mr-npc2a-3  Mr-npc2aORF-5  Mr-npc2aORF-3  Mr-npc2aORF-5  Mr-npc2aORF-3  Mr-npc2aORF-5  Mr-npc2aORF-3  Pgpd-5  Pgpd-3 | cggtaggatcccgcagaacgacggcca  ctggccgtccaagacgc  ctagctggccgtccaagacgc  gatcctggccgtccaagacgc  cggtaggatcccgcagaac  tgtcgtgccagctgcattaa  gcaattcggcgttaattcag  tttcgccagctggcgtaata  gagcttggatcagattgtcg  tctagaaaaaggtgacacaatacc  tctagatacaggtcacactgtcac  actagtgaaattttagctgtaatg  actagttttaagactcatcctgctc  aatttgcaagtcgtttcc  ctcatcggtcgacgattc  tctagaaacacgactgtatatgtag  tttaaagttgcagaccaaggtttc  tctagaaacacgactgtatatgtag  tctagacagtcatataattgcctc  atcgaattcgttatattcaaggactgtggaagcac atcgcggccgccttgatctccgccggaaac  ggatccatgcaaggctccctgctg  gatatctcacttgatctccgccg  gaattcatgcaaggctccctgctg  gatatccttgatctccgccg  gaattcgactgcccgcattgagaag  gaattcagatggaggagttggtgttg | Y-adaptor long chain (blunt end)  Y-adaptor short chain (blunt end)  Y-adaptor short chain (XbaI/SpeI/NheI end)  Y-adaptor short chain (BamHI and BglII end)  Adaptor primer used for exponent amplification  Cloning the left flanking sequence of T-DNA  Cloning the right flanking sequence of T-DNA  Disruption of *Mr-npc2a*  Confirmation of the Disruption of *Mr-npc2a*  Cloning the promoter region of *Mr-npc2a*  Cloning the genomic clone of *Mr-npc2a* for complementation  Cloning the ORF of *Mr-npc2a* for expression in *E. coli*  Cloning the ORF of *Mr-npc2a* for expression in *B. bassiana*  Cloning the ORF of *Mr-npc2a* for construction of the fusion protein Mr-NPC2a:GFP  Drive the expression the fusion protein Mr-NPC2a:GFP |
| GpdRT-5 | gactgcccgcattgagaag | RT-PCR analysis of *gpd* gene in *M. robertsii* |
| GpdRT-3 | agatggaggagttggtgttg |  |
| Rps3RT-5 | gtctggagcgcgcggttg | RT-PCR analysis of ribosome protein S3 |
| Rps3rt-3 | cactccttgcctgagaag |  |
